# Supplementary material for: Multicenter evaluation of label-free quantification in human plasma on a high dynamic range benchmark set
Source: Nat Commun. 2025 Oct 2;16:8774. doi: 10.1038/s41467-025-64501-z (PMC12491457; doi:10.1038/s41467-025-64501-z)
Supplement: Supplementary file 1 — Supplementary Information [file 41467_2025_64501_MOESM1_ESM.pdf]

1 Supplementary Figures

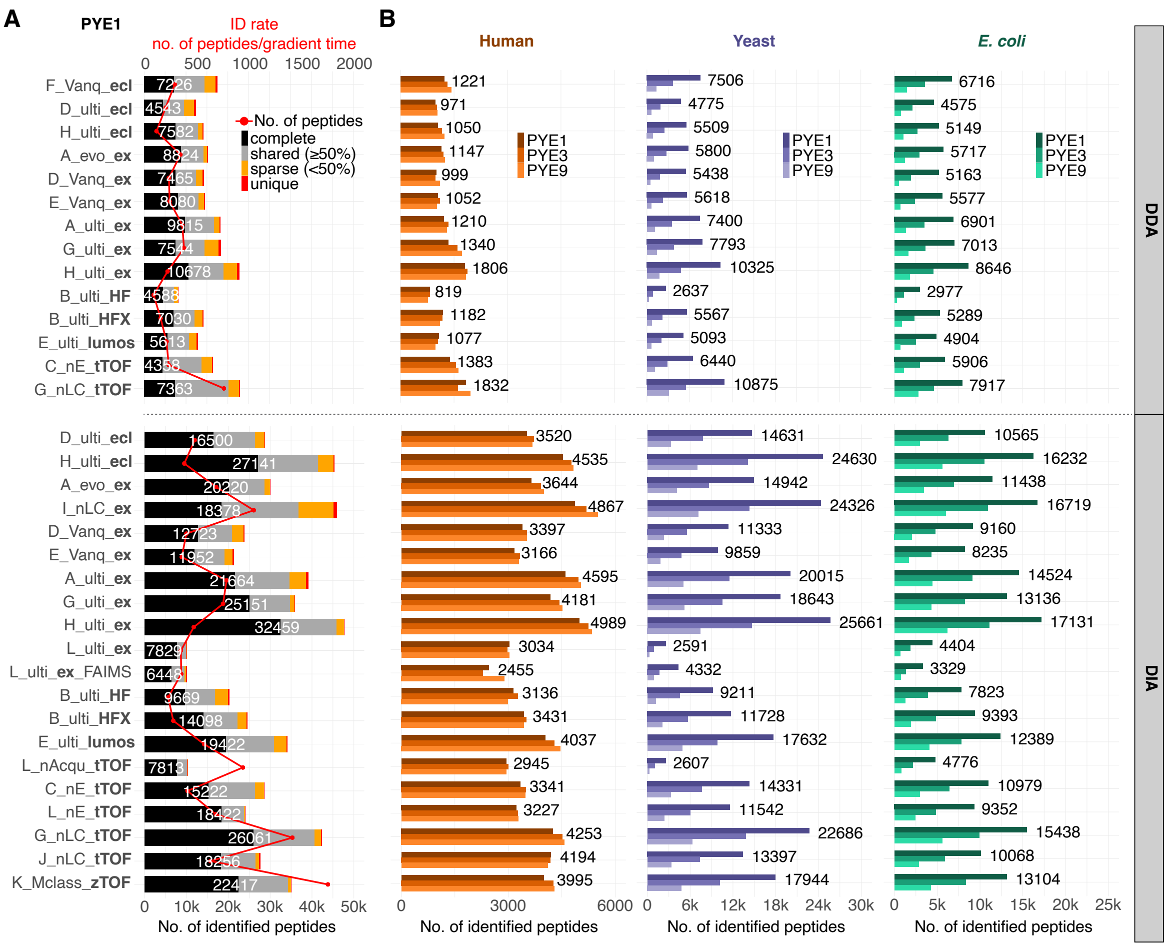

Supplementary Fig. 1. Number of identified peptides in the PYE sample set for different LC-MS setups and sites using MaxQuant (v2.3.1.0, DDA) and DIA-NN (v1.8.1, DIA) for data processing. (A) Number of identified peptides in the PYE1 sample set for the different setups. Colours indicate the number of peptides identified in all replicate runs per setup (complete, black), more than 50% of runs (grey) as well as sparse (below 50%, orange) and unique peptide identifications (red). White numbers indicate peptides identified in all replicate runs and red dots the number of identified peptides in relation to the programmed gradient length, i.e. peptide IDs per min. Letters refer to the laboratories. (B) Number of identified peptides across the whole PYE dataset (PYE1, PYE3, PYE9) for each setup split by species (human: orange, yeast: violet, *E. coli*: green). Numbers refer to peptides identified in PYE1. Source Data are provided as a Source Data file.

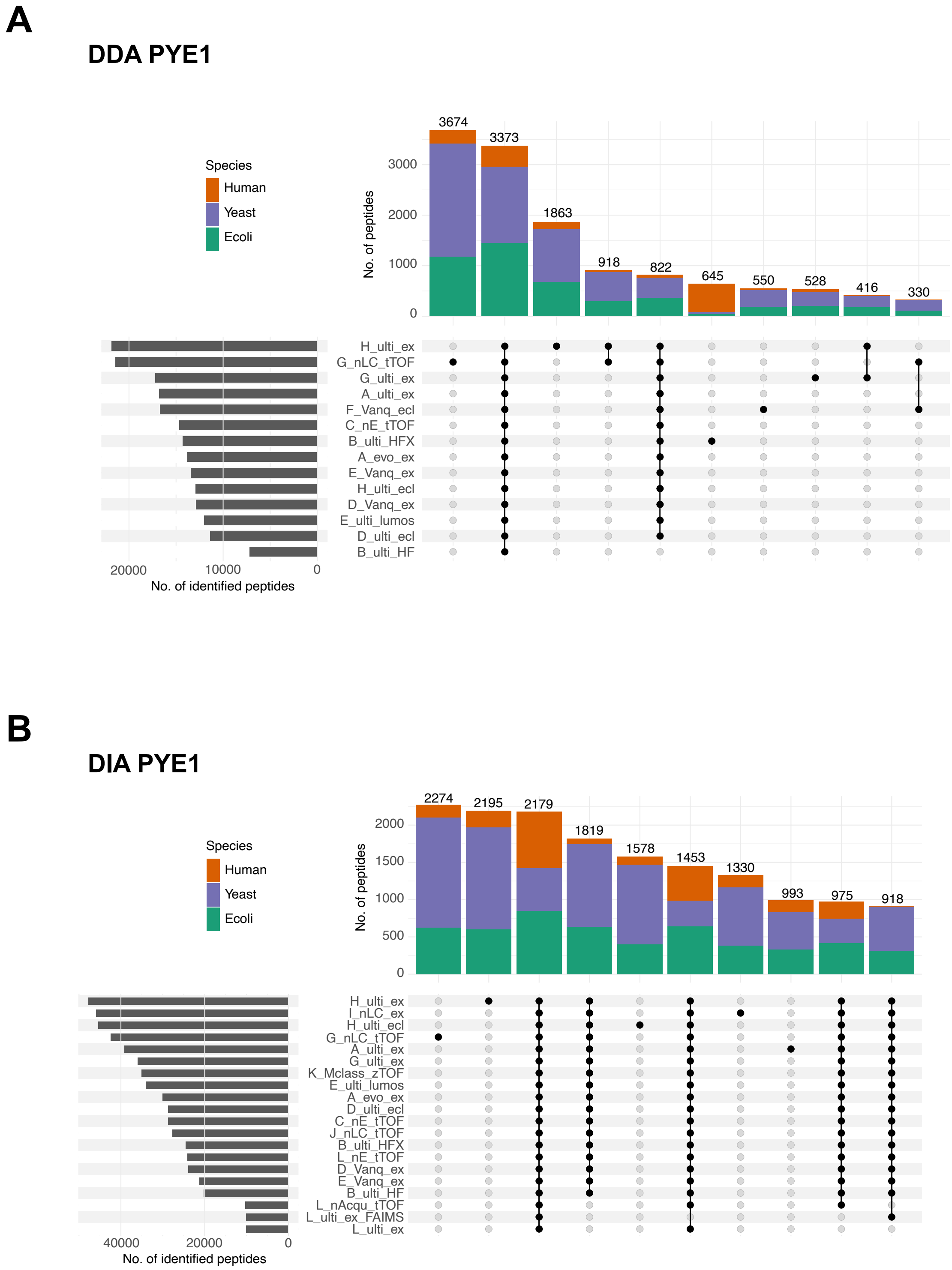

Supplementary Fig. 2. Overlap of peptides identified in the PYE1 sample set across multiple sites and LC-MS platforms using MaxQuant and DIA-NN for data processing. Upset plots display the overlap of identified peptides in the PYE1 sample set by (A) DDA-based and (B) DIA-based approaches. Data were processed with MaxQuant (v2.3.1.0, DDA) and DIA-NN (v1.8.1, DIA). Proportions of detected human (orange), yeast (violet) and *E. coli* (green) peptides are indicated in the bars.

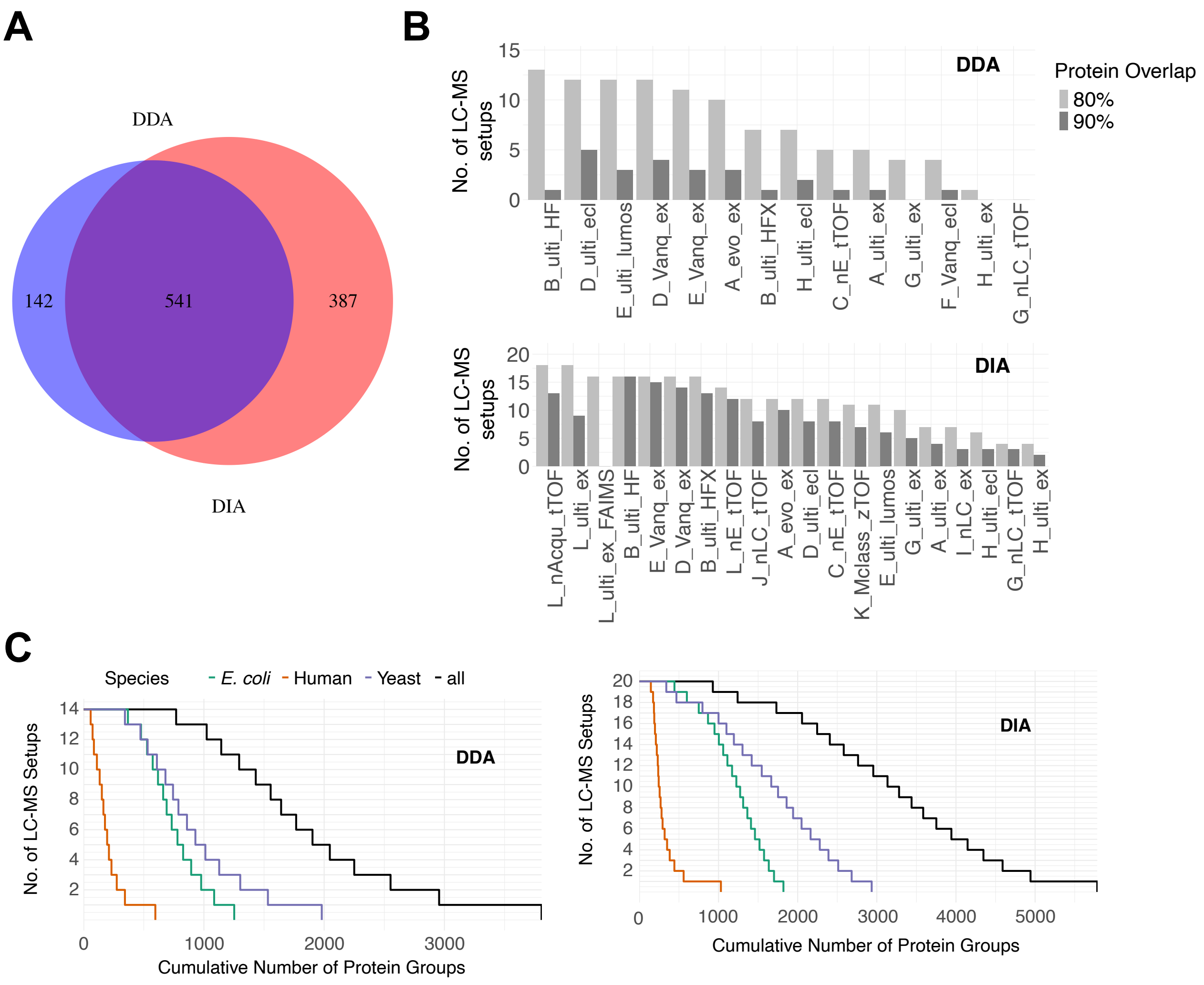

Supplementary Fig. 3. Summary statistics on the numbers of proteins identified across all 34 LC-MS setups. Data processing was conducted using MaxQuant (v2.3.1.0, DDA) and DIA-NN (v1.8.1, DIA). (A) In total, 541 proteins were shared and commonly identified in all 34 LC-MS setups. To generate the Venn diagram, only those proteins were selected that were identified across all 14 DDA as well as all 20 DIA LC-MS setups (see also Figure 2 C,D). (B) Count of LC-MS setups that share 80% (light grey) and 90% (dark grey) of proteins with the respective setup listed on the x-axis. Particularly, for DIA-based approaches the number of setups with e.g. similar proteome coverage or setups with deep coverage that identify >80 % or even >90 % of proteins from setups with lower IDs is high indicating good identification reproducibility across multiple platforms and sites. (C) Number of proteins that are identified across multiple LC-MS setups split by species (human: orange, *E. coli*: green, yeast: violet, black: sum of all proteins). Source data are provided as a Source Data file.

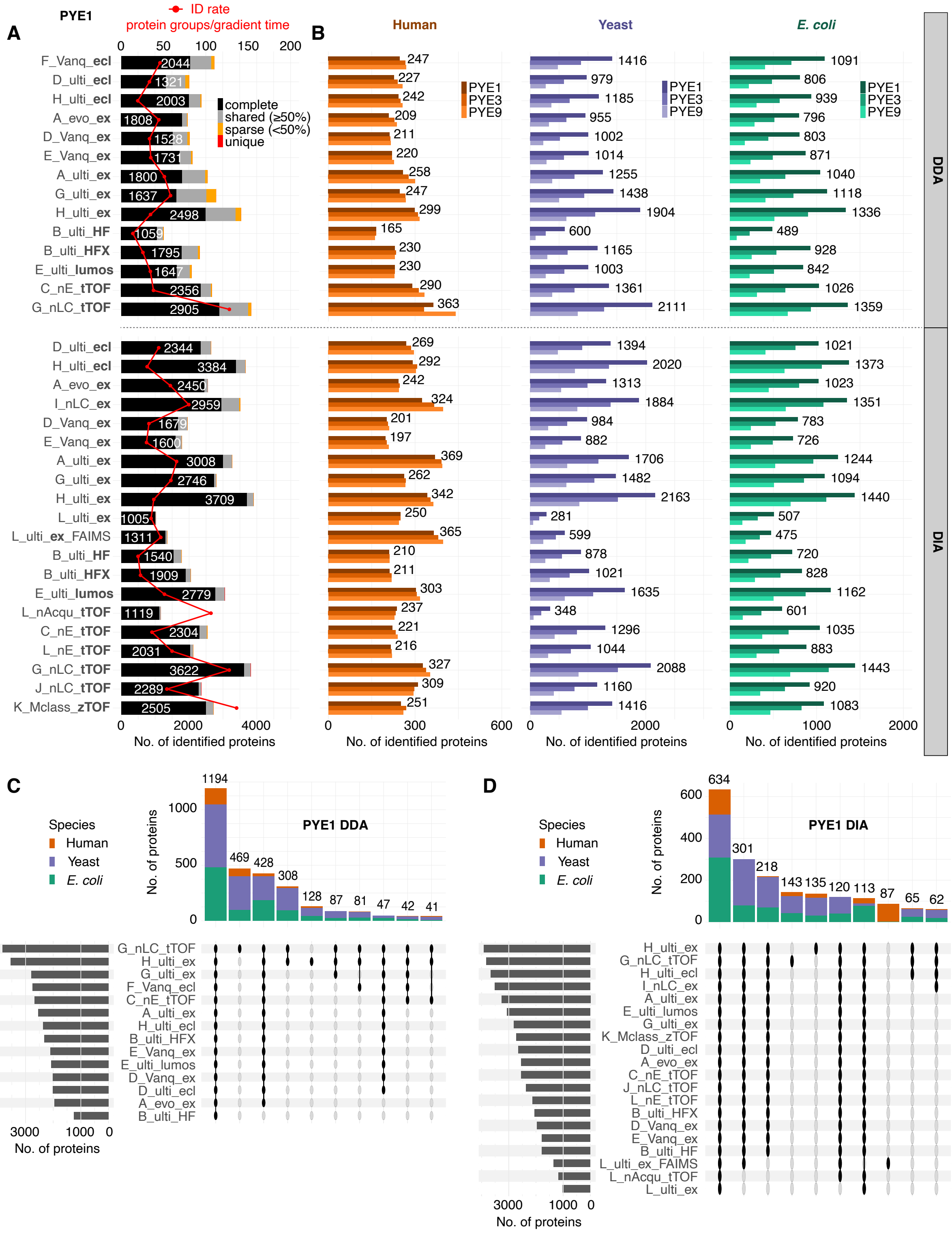

Supplementary Fig. 4. Number of identified proteins in the PYE sample set for different LC-MS setups and sites using FragPipe (v23.0, DDA and DIA) for data processing. (A) Number of identified protein groups in the PYE1 sample set for the different setups. Colours indicate the number of proteins identified in all replicate runs per setup (complete, black), more than 50% of runs (grey) as well as sparse (below 50%, orange) and unique identifications (red). White numbers indicate proteins

identified in all replicate runs and red dots the number of identified proteins in relation to the programmed gradient length, i.e. protein IDs per min. Letters refer to the laboratories. (B) Number of identified protein groups across the whole PYE dataset (PYE1, PYE3, PYE9) for each setup split by species (human: orange, yeast: violet, *E. coli*: green). Numbers refer to proteins identified in PYE1. (C,D) Upset plots showing the overlap of identified proteins in the PYE1 sample set by (C) DDA- and (D) DIA-based approaches across multiple sites and LC-MS platforms. Proportions of human (orange), yeast (violet) and *E. coli* (green) proteins are indicated within the bars. Source data are provided as a Source Data file.

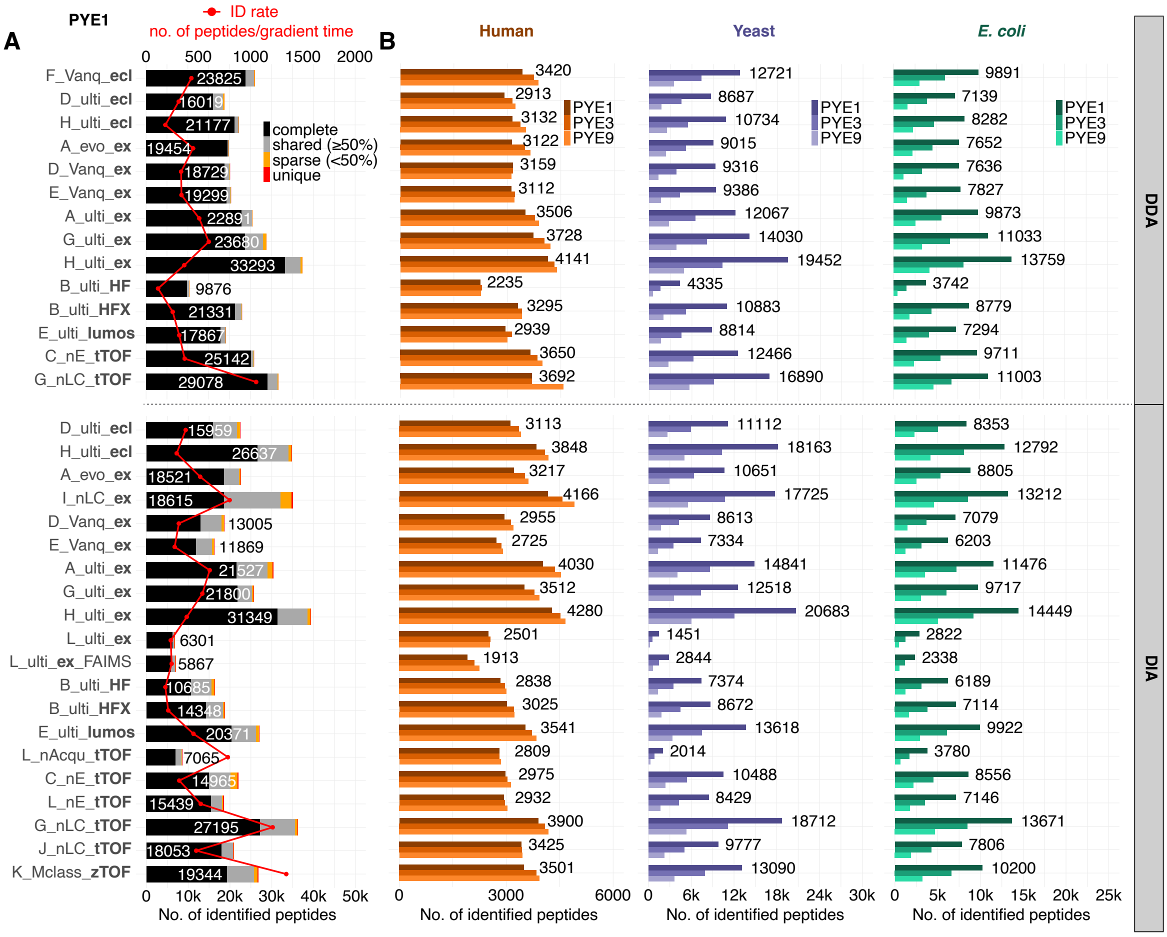

Supplementary Fig. 5. Number of identified peptides in the PYE sample set for different LC-MS setups and sites using FragPipe (v23.0, DDA and DIA) for data processing. (A) Number of identified peptides in the PYE1 sample set for the different setups. Colours indicate the number of peptides identified in all replicate runs per setup (complete, black), more than 50% of runs (grey) as well as sparse (below 50%, orange) and unique peptide identifications (red). White numbers indicate peptides identified in all replicate runs and red dots the number of identified peptides in relation to the programmed gradient length, i.e. peptide IDs per min. Letters refer to the laboratories. (B) Number of identified peptides across the whole PYE dataset (PYE1, PYE3, PYE9) for each setup split by species (human: orange, yeast: violet, *E. coli*: green). Numbers refer to peptides identified in PYE1. Source data are provided as a Source Data file.

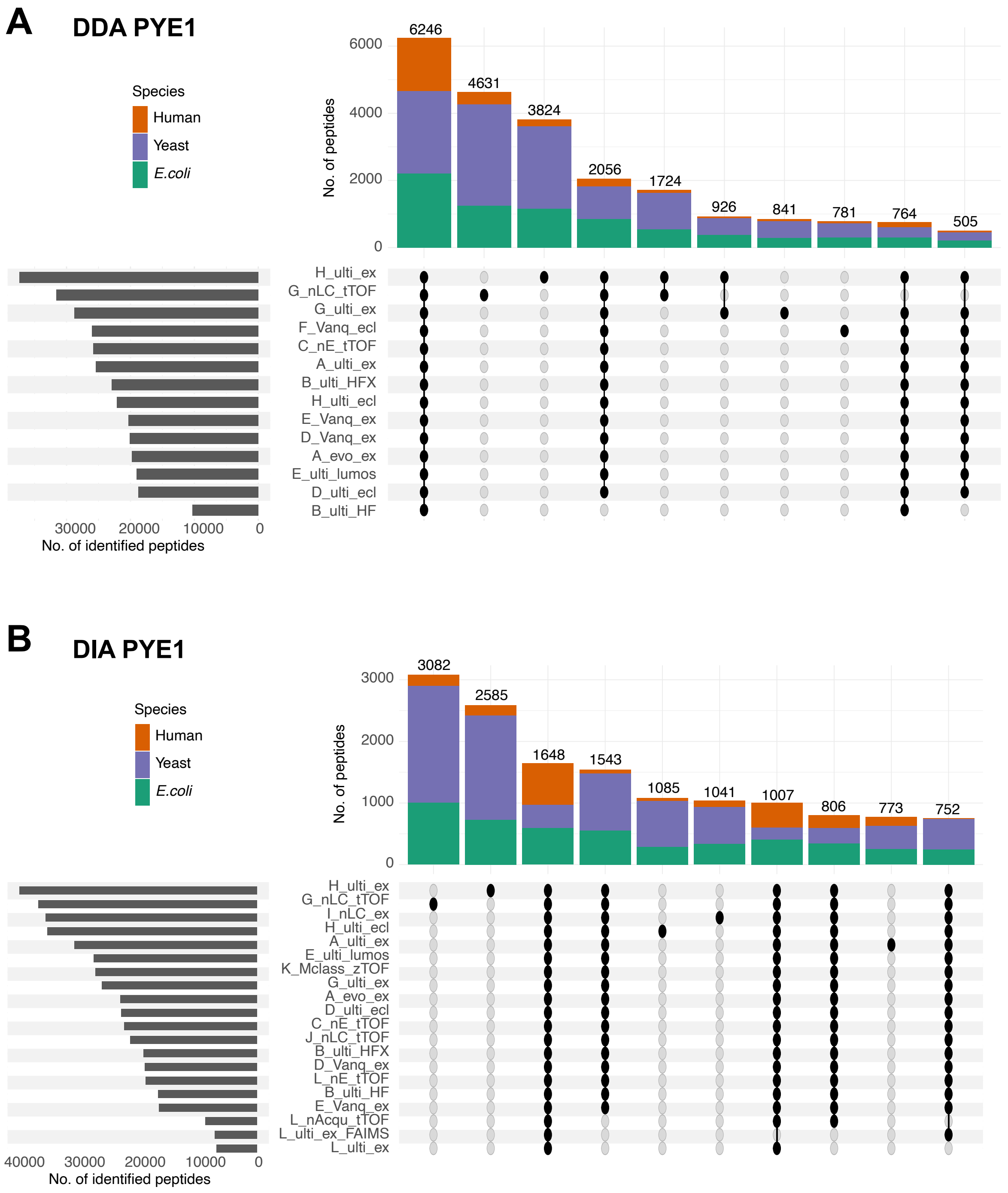

Supplementary Fig. 6. Overlap of peptides identified in the PYE1 sample set across multiple sites and LC-MS platforms using FragPipe for data processing. Upset plots display the overlap of identified peptides in the PYE1 sample set by (A) DDA-based and (B) DIA-based approaches. Data were processed with FragPipe v23.0 (DDA and DIA). Proportions of detected human (orange), yeast (violet) and *E. coli* (green) peptides are indicated in the bars.

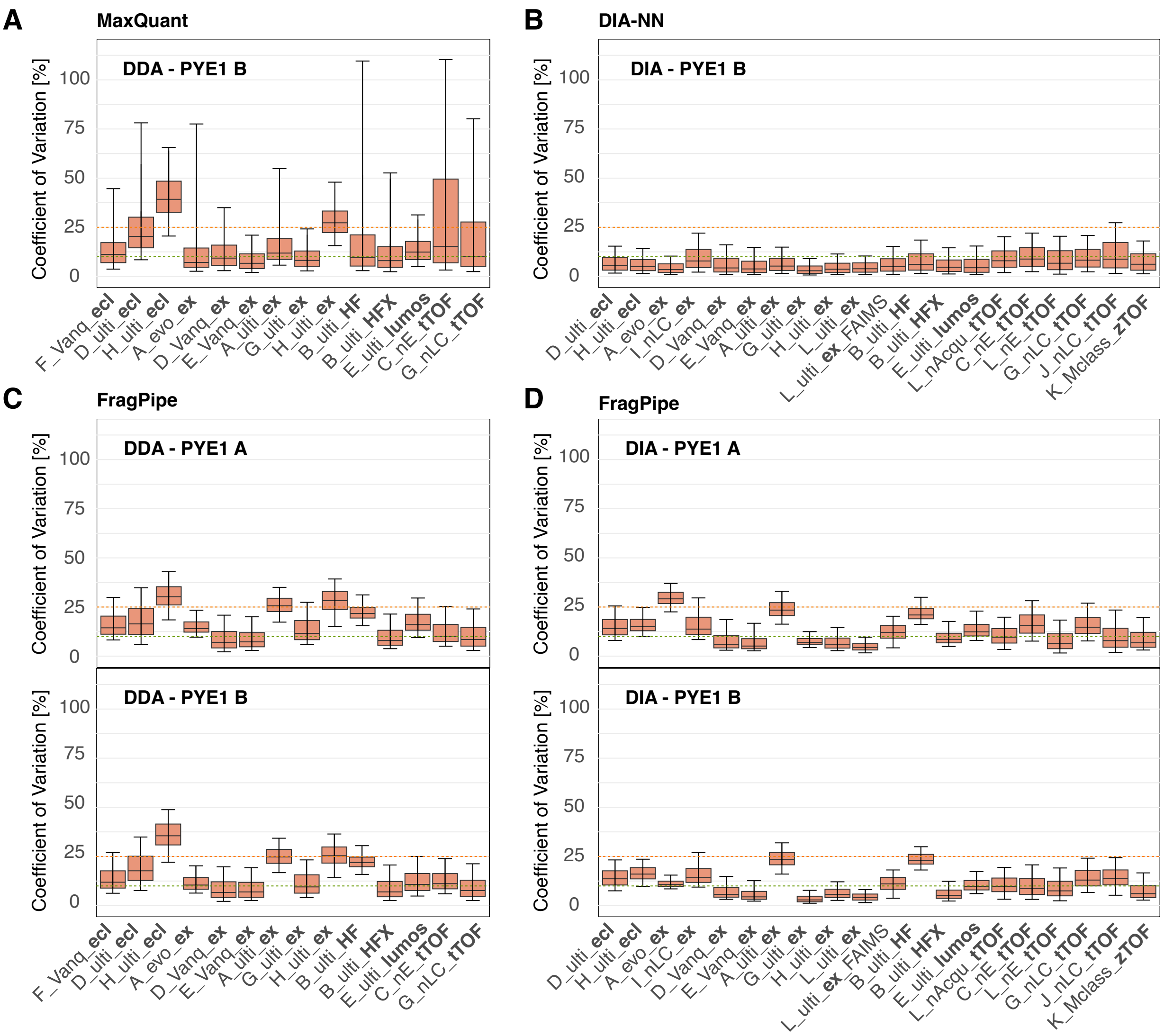

Supplementary Fig. 7. Reproducibility of LC-MS analyses. Coefficients of variation (CVs) of protein abundances for replicate analyses of sample PYE1 B were calculated for each LC-MS setup. MaxQuant (v2.3.1.0) was used for (A) DDA data processing and DIA-NN (v1.8.1) for DIA data analysis. (C, D) CVs of protein abundances for replicate analyses of samples PYE1 A (upper) and PYE1 B (lower panel) calculated for each LC-MS setup after data processing with FragPipe v23.0. Panel (C) displays CVs for the DDA and panel (D) for the DIA data. Processing of DDA data with the latest version of FragPipe (v23.0) resulted in markedly lower CVs as compared to MaxQuant. Nevertheless, both DDA data sets (A, C) show lower quantitative reproducibility as compared to the DIA data (B, C). Boxplot center lines represent the median value, boundaries the interquartile range and whiskers the 5th/90th percentiles of the dataset. The red line marks 25% CV and green line 10% CV. For detailed information on the number of replicate injections for each setup (*n* = 6 in most cases) see Table 1. Source data are provided as a Source Data file.

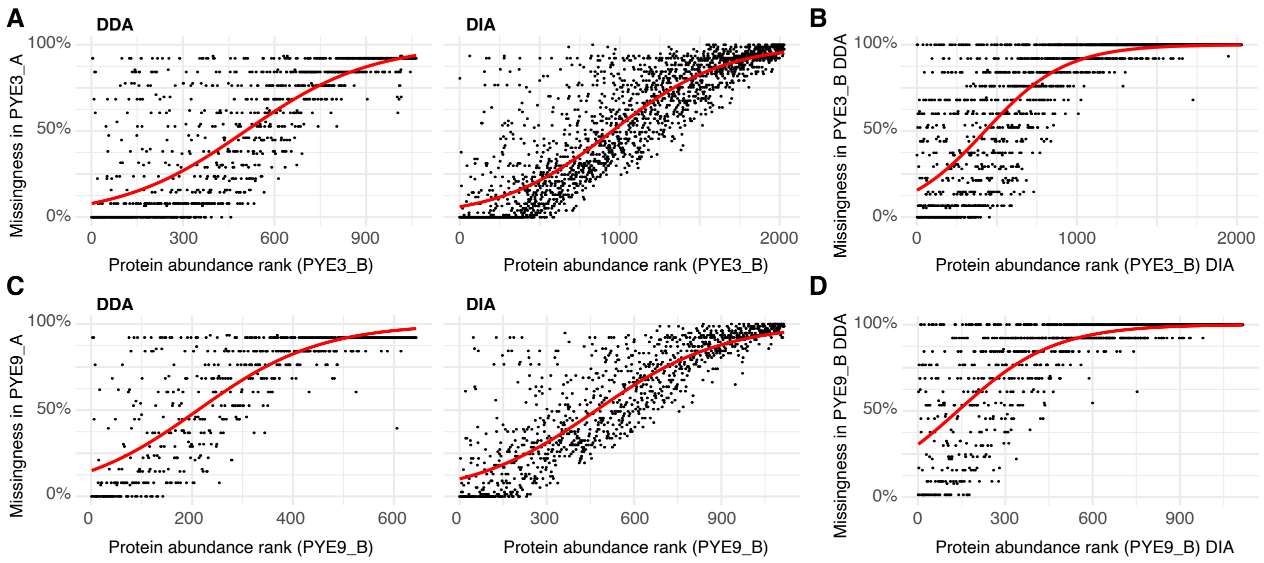

Supplementary Fig. 8. Comparison of missing values in the DDA versus the DIA dataset for PYE3 and PYE9 demonstrate higher proteome coverage and less missingness in the DIA dataset. Missing values were compared between the 13 DDA and DIA data sets that were acquired with the exact same LC-MS setup, see Table 1 and Supplementary Data 1. (A) Percent of missing values for yeast proteins in PYE3 A as compared to PYE3 B (ranked by protein abundance) for the DDA and DIA dataset. (B) Percent of missing values for yeast proteins in the PYE3 B DDA dataset as compared to the PYE3 B DIA dataset dependent on protein abundance. (C) Percent of missing values for yeast proteins in PYE9 A as compared to PYE9 B (ranked by protein abundance) for the DDA and DIA dataset. (D) Percent of missing values for yeast proteins in the PYE9 B DDA dataset as compared to the PYE9 B DIA dataset dependent on protein abundance. X-axis: Rank as defined by the average normalized intensity ($INT_{Protein}/INT_{max}$) across all 13 setups. Y-axis: Missingness (1-(number of detections/number of runs)) across all 13 setups and injection replicates as percent values.

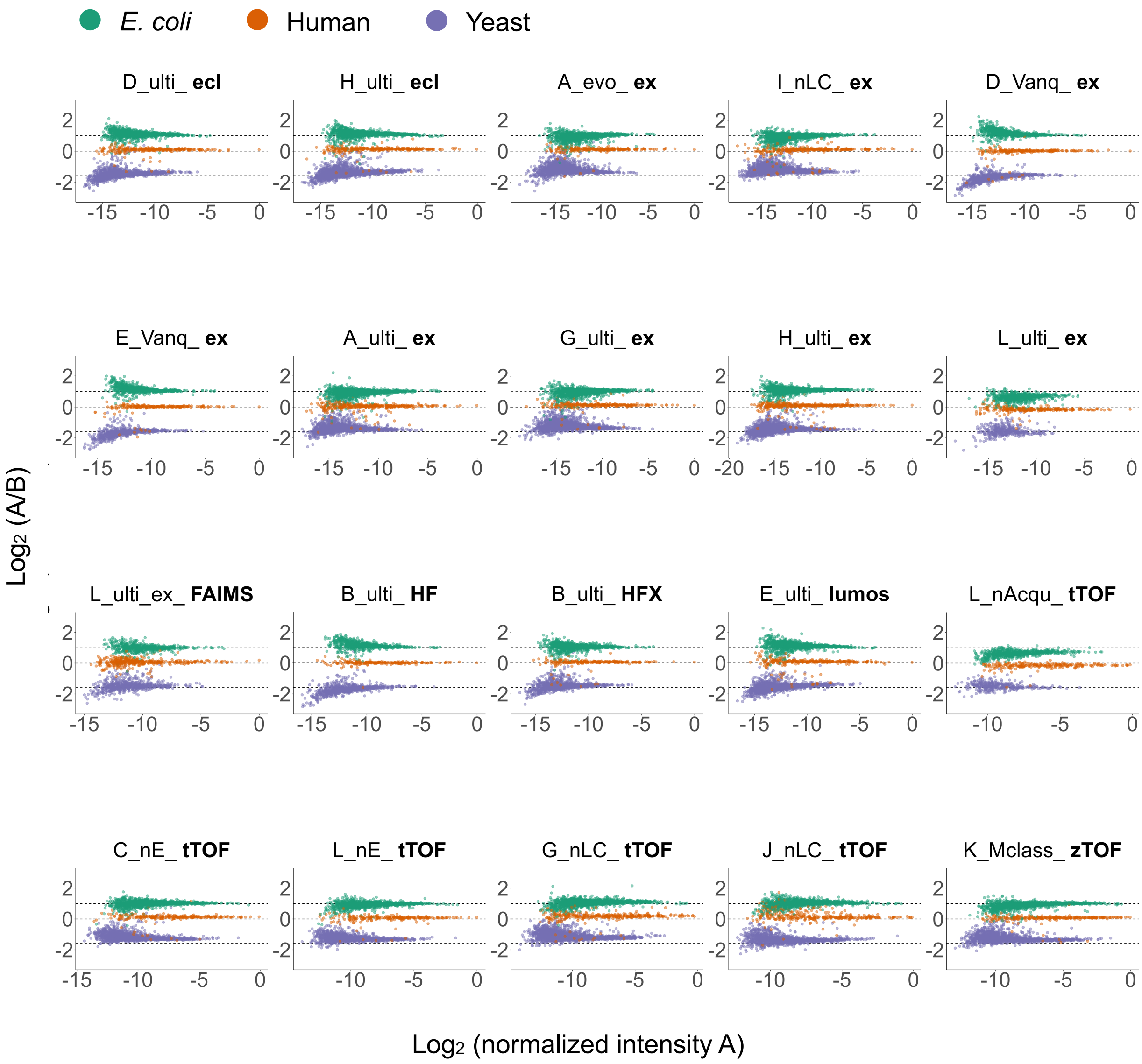

Supplementary Fig. 9. Protein level label-free quantification results for the PYE1 set analysed with different LC-MS setups across multiple sites in DIA mode. Log-transformed ratios ($\log_2$(A/B)) of proteins were plotted over the log-transformed intensity of sample A for the PYE1 set summarizing the quantitative results for the DIA data acquired with 20 different LC-MS setups. Data were processed with DIA-NN (v1.8.1).

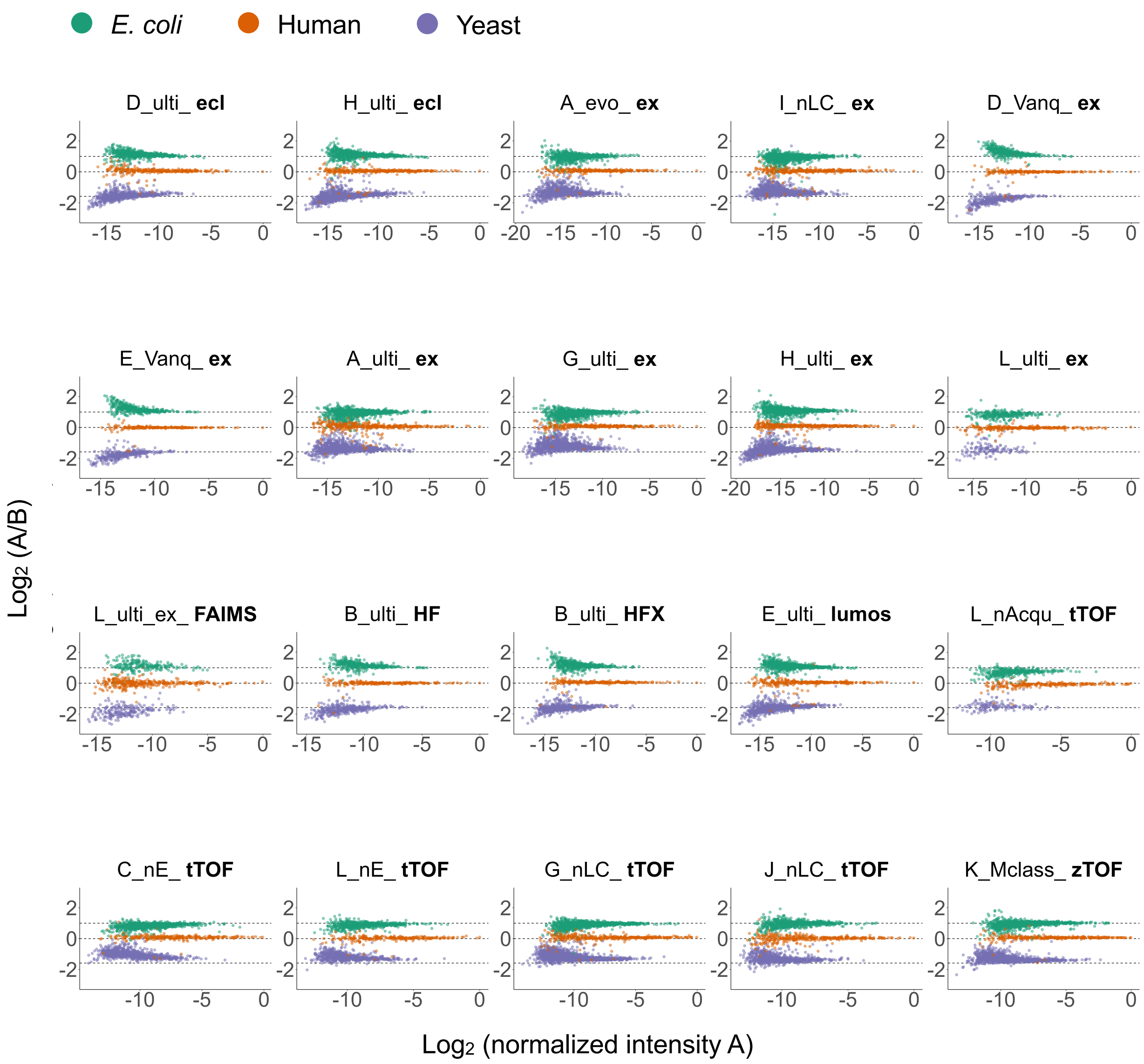

Supplementary Fig. 10. Protein level label-free quantification results for the PYE3 set analysed with different LC-MS setups across multiple sites in DIA mode. Log-transformed ratios (log_2(A/B)) of proteins were plotted over the log-transformed intensity of sample A for the PYE3 set summarizing the quantitative results for the DIA data acquired with 20 different LC-MS setups. Data were processed with DIA-NN (v1.8.1).

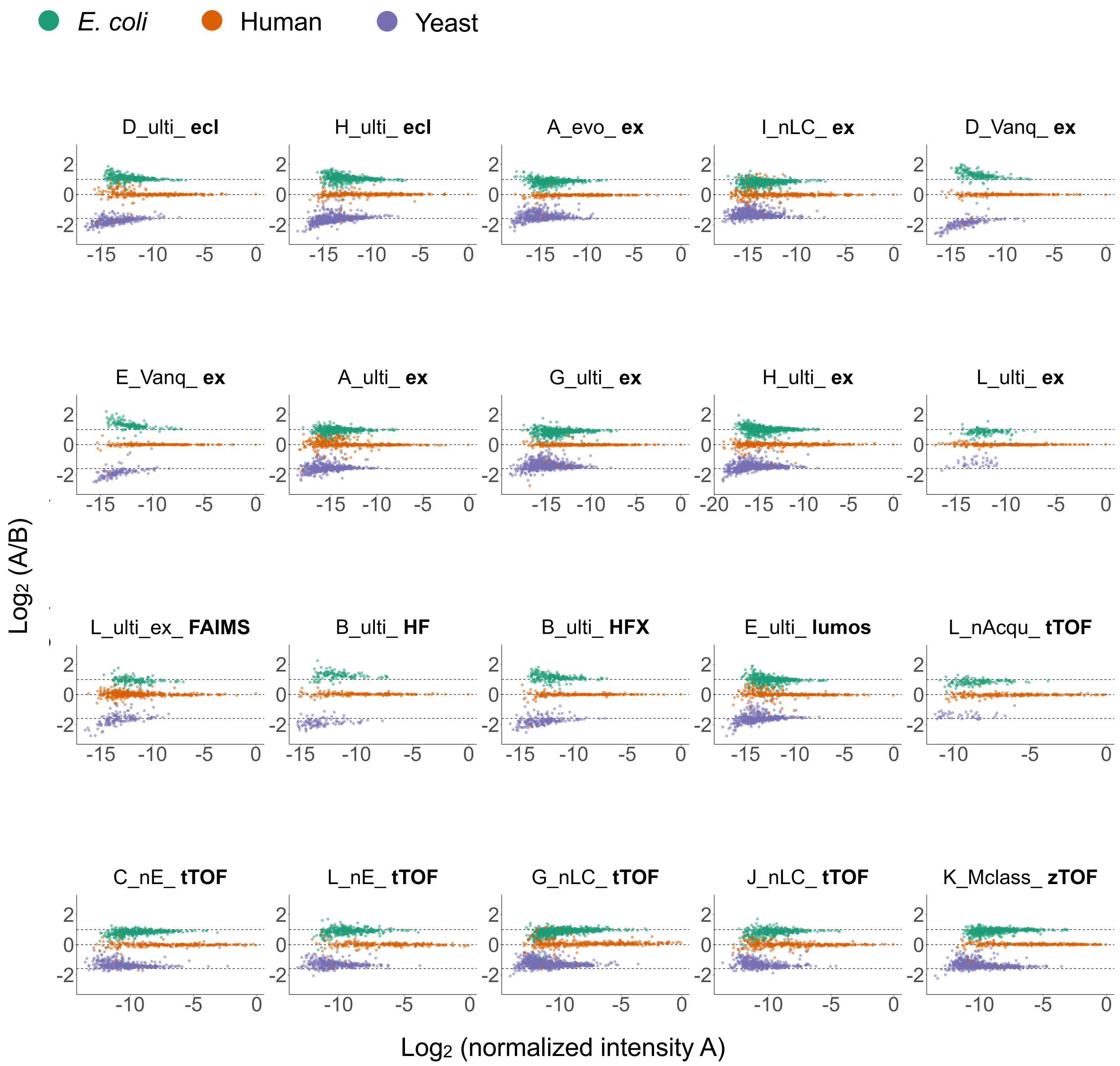

Supplementary Fig. 11. Protein level label-free quantification results for the PYE9 set analysed with different LC-MS setups across multiple sites in DIA mode. Log-transformed ratios ($\log_2$(A/B)) of proteins were plotted over the log-transformed intensity of sample A for the PYE9 set summarizing the quantitative results for the DIA data acquired with 20 different LC-MS setups. Data were processed with DIA-NN (v1.8.1).

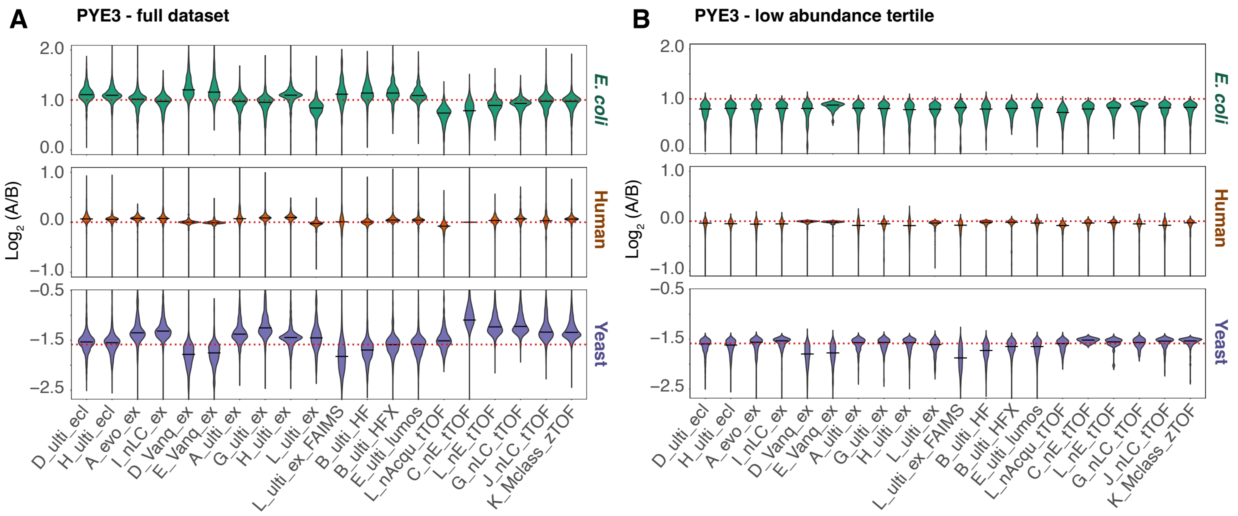

Supplementary Fig. 12. Quantitative metrics of the DIA dataset. Violin plots of log-transformed ratios ($\log_2$(A/B)) of protein abundances for the (A) full PYE3 set as well as (B) for the low abundance tertile. Data were acquired in DIA mode with 20 different LC-MS setups. Solid lines within the violin plot indicate the median $\log_2$(A/B) value for each setup and dashed red lines the expected $\log_2$(A/B) values for human (orange), yeast (violet), and *E. coli* (green) proteins. Data were processed with DIA-NN (v1.8.1).

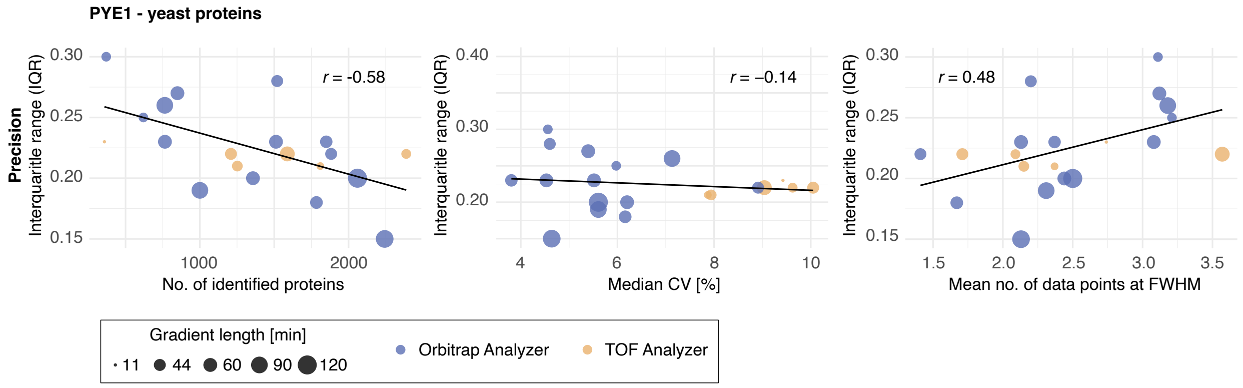

Supplementary Fig. 13. Number of quantified proteins is one of the main factors affecting precision. Correlation of precision (defined as interquartile range Q075 – Q025, calculated for yeast proteins in the PYE1 set) with other metrics such as number of identified proteins (left), average of median CVs [%] of protein abundances in samples PYE1A and PYE1B (middle) and number of datapoints acquired across the chromatographic peak (right panel). Dot sizes indicate gradient length. Blue: Orbitrap, orange: TOF analyzer, see also Supplementary Data 7. Source data are provided as a Source Data file.

2 Extended Methods: LC-MS settings

2.1 Laboratory A

DDA - UltiMate 3000 RSLC & Exploris 480:

300 ng (1.5 µL) of PYE peptide mixture was injected to an UltiMate 3000 RSLC nano-HPLC (Dionex, Germering, Germany) coupled to an Exploris 480 mass spectrometer (Thermo Fisher Scientific, Bremen, Germany). Peptides were separated on an IonOpticks Odyssey column (25 cm x 75 µm, C18 1.6 µm) at 40°C by a 50 minutes non-linear gradient at a flow rate of 300 nL/min. Data were acquired in a data-dependent mode with a Top20 method. MS1 spectra were acquired with a resolution of 60,000 in a mass range from *m/z* 350-1,200 with 300% AGC target and a maximum injection time of 25 ms. The resolution for MS2 was 15,000, AGC target set to standard, and maximum injection time to 22 ms. Fragment ion data were collected in centroid mode. Normalized HCD collision energy was 30%. First mass was defined at *m/z* 110. Isolation width was set to 1.4 Th.

DDA - Evosep One & Exploris 480:

The Evotips for each run were loaded with 20 µL peptide solution containing around 500 ng of sample peptides. They were placed in the Evosep One (Evosep, Odense, Denmark) autosampler until analysis. The 30 samples per day method employing a 44 minutes gradient was chosen and a 15 cm column (Dr. Maisch C18 AQ, 1.5 µm beads, 150 µm ID) used for separation of peptides. The LC was coupled to an Exploris 480 mass spectrometer (Thermo Fisher Scientific, Bremen, Germany). Data were acquired in a data-dependent mode with a Top20 method. MS1 spectra were acquired with a resolution of 120,000 in a mass range from *m/z* 375-1,500. The resolution for MS2 was 15,000, AGC target 100% and maximum injection time 40 ms. Fragment ion data were recorded in profile mode. Isolation width was set to 1.4 Th.

DIA - UltiMate 3000 RSLC & Exploris 480:

300 ng (1.5 µL) of PYE peptide mixture was injected to an UltiMate 3000 RSLC nano-HPLC (Dionex, Germering, Germany) coupled to an Exploris 480 mass spectrometer (Thermo Fisher Scientific, Bremen, Germany). Peptides were separated on an IonOpticks Odyssey column (25 cm x 75 µm, C18 1.6 µm) at 40°C by a 50 minutes

non-linear gradient at a flow rate of 300 nL/min. Data-independent acquisition was performed at a full scan resolution of 120,000. MS1 spectra were recorded over a mass range from *m/z* 380-980, maximum injection time was set to 100 ms and the normalized AGC target to 300%. The MS1 scan was followed by DIA scans covering a precursor mass range between *m/z* 380-980 at an isolation window width of 20 Th with 1 Th overlap. Thus, the number of scan events was 30. The resolution was set to 30,000 and the AGC target to 3,000%.

<u>DIA - Evosep One & Exploris 480:</u>
The Evotips for each run were loaded with 20 µL peptide solution containing around 500 ng of sample peptides. They were placed in the Evosep One (Evosep, Odense, Denmark) autosampler until analysis. The 30 samples per day method employing a 44 minutes gradient was chosen and a 15 cm column (Dr. Maisch C18 AQ, 1.5 µm beads, 150 µm ID) used for separation of peptides. The LC was coupled to an Exploris 480 mass spectrometer (Thermo Fisher Scientific, Bremen, Germany). Data-independent acquisition was performed at a full scan resolution of 120,000. MS1 spectra were recorded over a mass range from *m/z* 380-980, maximum injection time was set to 100 ms and the normalized AGC target to 300%. The MS1 scan was followed by DIA scans covering a precursor mass range between *m/z* 380-980 at an isolation window width of 20 Th with 1 Th overlap. Thus, the number of scan events was 30. The resolution was set to 30,000 and the AGC target to 3,000%.

2.2 Laboratory B

<u>DDA - UltiMate 3000 RSLC & Q Exactive HF:</u>
The peptide mixtures, prepared as stated in the instruction, were injected into an UltiMate 3000 RSLC nano-HPLC (Thermo Fisher Scientific, Bremen, Germany) coupled to a Q Exactive HF mass spectrometer (Thermo Fisher Scientific). Peptides were separated on a nanoEase M/Z HSS T3 column (25 cm x 75 µm, C18 1.8 µm, 100 Å, Waters, Eschborn, Germany) at 40°C by a 90 minutes non-linear gradient at a flow rate of 250 nL/min. Data were acquired in a data-dependent mode. MS1 spectra were recorded at a resolution of 60,000 in a mass range from *m/z* 300 to 1,500. From the MS1 scan, the 10 most abundant ions were selected for HCD fragmentation with a normalized collision energy of 27, an isolation window width of 1.6 Th, and a dynamic

exclusion of 30 s. The resolution for MS2 was 15,000, the AGC target was set to 1e5 and maximum injection time to 50 ms. Unassigned charges, and charges of “1 plus” and “>8 plus” were excluded. Spectra were recorded in profile mode.

DDA - UltiMate 3000 RSLC & Q Exactive HFX:

The peptide mixtures, prepared as stated in the instruction, were injected to an UltiMate 3000 RSLC nano-HPLC (Dionex, Germering, Germany) coupled to a Q Exactive HFX mass spectrometer (Thermo Fisher Scientific, Bremen, Germany). Tryptic peptides were automatically loaded on a C18 trap column (300 µm inner diameter (ID) × 5 mm, Acclaim PepMap100 C18, 5 µm, 100 Å, LC Packings) at 30 µL/min flow rate, followed by separation on a C18 reversed phase analytical column (nanoEase M/Z HSS T3 column; 25 cm x 75 µm, C18 1.8 µm, 100 Å, Waters, Eschborn, Germany) at 40°C by a 90 minutes non-linear gradient at a flow rate of 250 nL/min. Data were acquired in a data-dependent mode with a Top15 method if peptide ions were at least doubly charged. The high-resolution (60,000 full width at half-maximum) MS spectrum was acquired with a mass range from *m/z* 300 to 1,500 with automatic gain control target (AGC) set to 3 x 10^6 and a maximum of 30 ms injection time. The resolution for MS2 was 15,000, the AGC target was set to 1e5 and the maximum injection time to 50 ms. Isolation window width was 1.6 Th. The normalized collision energy was 28, and the spectra were recorded in profile mode.

DIA - UltiMate 3000 RSLC & Q Exactive HF:

The peptide mixtures, prepared as stated in the instruction, were injected to an UltiMate 3000 RSLC nano-HPLC (Dionex, Germering, Germany) coupled to a Q Exactive HF mass spectrometer (Thermo Fisher Scientific, Bremen, Germany). Peptides were separated on a nanoEase M/Z HSS T3 column (25 cm x 75 µm, C18 1.8 µm, 100 Å) at 40°C by a 90 minutes non-linear gradient at a flow rate of 250 nL/min. The DIA method consisted of a survey scan from *m/z* 300 to 1,650 at 120,000 resolution and an automatic gain control (AGC) target of 3e6 or 120 ms maximum injection time. Fragmentation was performed via higher energy collisional dissociation with a target value of 3e6 ions determined with predictive AGC. Precursor peptides were isolated with 37 variable windows spanning a precursor mass range from *m/z* 300 to 1650 at 30,000 resolution with an AGC target of 3e6 and automatic injection

time. The normalized collision energy was 28, and the spectra were recorded in profile mode.

DIA - UltiMate 3000 RSLC & Q Exactive HFX:

The peptide mixture, prepared as stated in the instruction, were injected to an UltiMate 3000 RSLC nano-HPLC (Dionex, Germering, Germany) coupled to a Q Exactive HFX mass spectrometer (Thermo Fisher Scientific, Bremen, Germany). Peptides were separated on a nanoEase M/Z HSS T3 column (25 cm x 75 µm, C18 1.8 µm, 100 Å) at 40°C by a 90 minutes non-linear gradient at a flow rate of 250 nL/min. The DIA method consisted of a survey scan from *m/z* 300 to 1,650 at 120,000 resolution and an automatic gain control (AGC) target of 3e6 or 120 ms maximum injection time. Fragmentation was performed via higher energy collisional dissociation with a target value of 3e6 ions determined with predictive AGC. Precursor peptides were isolated with 37 variable windows spanning from *m/z* 300 to 1,650 at 30,000 resolution with an AGC target of 3e6 and automatic injection time. The normalized collision energy was 28, and the spectra were recorded in profile mode.

2.3 Laboratory C

DDA - nanoElute & timsTOF Pro:

Samples were analyzed on a nanoElute LC coupled to a timsTOF Pro mass spectrometer with a CaptiveSpray ion source (Bruker Corporation, Billerica, MA, USA). Samples were injected on a 15 cm self-packed C18 column (75 µm internal diameter) with 1.9 µm ReproSil-Pur 120 C18-AQ resin (Dr Maisch, Germany). A gradient of water (A) and acetonitrile (B) supplemented with 0.1% formic acid was applied at a flow rate of 300 nL/min and a column temperature of 50°C. The following gradient was applied: 0 min, 2%B; 2 min, 5%B; 62 min, 24%B; 72 min, 35%B; 75 min, 60%B; 78 min, 85%B. The MS was operated in data-dependent acquisition parallel accumulation-serial fragmentation (ddaPASEF) mode. Ion accumulation and separation using trapped ion mobility spectrometry (TIMS) was set to a ramp time of 100 ms with an ion mobility range ($1/K_0$) from 0.6 to 1.6 Vs/cm^2. One MS cycle included one TIMS full MS scan and 10 PASEF scans with up to 12 precursors targeted per scan.

DIA - nanoElute & timsTOF Pro:

Samples were analyzed on a nanoElute LC coupled to a timsTOF Pro mass spectrometer with a CaptiveSpray ion source (Bruker Corporation, Billerica, MA, USA). Samples were injected on a 15 cm self-packed C18 column (75 µm internal diameter) with 1.9 µm ReproSil-Pur 120 C18-AQ resin (Dr Maisch, Germany). A gradient of water (A) and acetonitrile (B) supplemented with 0.1% formic acid was applied at a flow rate of 300 nL/min and a column temperature of 50°C. The following gradient was applied: 0 min, 2%B; 2 min, 5%B; 62 min, 24%B; 72 min, 35%B; 75 min, 60%B; 78 min, 85%B. The MS was operated in a data-independent acquisition parallel accumulation-serial fragmentation (diaPASEF) mode. Ion accumulation and separation using trapped ion mobility spectrometry (TIMS) was set to a ramp time of 100 ms with an ion mobility range ($1/K_0$) from 0.6 to 1.45 Vs/cm^2. One MS cycle included one TIMS full MS scan followed by multiple MS/MS scans comprising in total 26 windows with a width of 27 Th covering a *m/z*-range of 350 to 1,002. Two windows were recorded per PASEF scan. This resulted in a total cycle time of 1.4 s.

2.4 Laboratory D

<u>DDA – Vanquish Neo & Exploris 480:</u>

Mixed proteome samples (2 µg/µL protein, prepared in the Tenzer lab, based on the concept initially described in Navarro *et al.*, 2016[1]) containing tryptic digest of human plasma with variable spike-ins of tryptically digested yeast and *E.coli*, respectively, were transferred undiluted to MS vials. Samples were analysed in five replicate injections according to the following measurement scheme: blank-500 ng in-house Hela QC-2x blank-six samples (A9,B9,A3,B3,A1,B1)-blank.

Peptides were analyzed on a Vanquish Neo (micro-flow configuration) coupled on-line to an Orbitrap Exploris 480 mass spectrometer (Thermo Fisher Scientific, Bremen, Germany). 10 µg of peptides were applied onto a commercially available Acclaim PepMap 100 C18 column (2 µm particle size, 1 mm ID × 150 mm, 100 Å pore size; Thermo Fisher Scientific), and separated using a two-stepped gradient: In the first step, a 50 minutes linear gradient ranging from 3% to 24% solvent B (0.1% FA, 3% DMSO in ACN) in solvent A (0.1% FA, 3% DMSO in HPLC grade water) at a flow rate of 50 µL/min was applied. In the second step, solvent B was further increased from 24% to 31% over a 10 minute linear gradient. The mass spectrometer was operated in data-dependent acquisition (DDA) and positive ionization mode. MS1 full scans (*m/z* 360 –

1,300) were acquired with a resolution of 60,000, a normalized AGC value of 100% and a maximum injection time of 25 ms. Peptide precursor selection for fragmentation was carried out using a fixed cycle time of 1.2 s. Only precursors with charge states from 2 to 6 were selected and dynamic exclusion of 30 s was enabled. Peptide fragmentation was performed using higher energy collision induced dissociation (HCD) and a normalized collision energy of 28%. The precursor isolation window width of the quadrupole was set to 1.3 Th. MS2 spectra were acquired with a resolution of 15,000, a fixed first mass of *m/z* 100, a normalized automatic gain control (AGC) target value of 100% and maximum injection time of 22 ms.

<u>DDA – UltiMate 3000 RSLC & Orbitrap Eclipse:</u>

Mixed proteome samples (2 µg/µL protein, prepared in the Tenzer lab, based on the concept initially described in Navarro et al., 2016[1]) containing tryptic digest of human plasma with variable spike-ins of tryptically digested yeast and *E.coli*, respectively, were diluted 33.3-fold with 0.1% formic acid and transferred to MS vials. Samples were analysed in six replicate injections according to the following measurement scheme: blank-100 ng in-house Hela QC-2x blank-six samples (A9,B9,A3,B3,A1,B1)-blank.
LC-MS/MS measurements were performed on a Dionex Ultimate 3000 RSLC nano system coupled to an Orbitrap Eclipse mass spectrometer (Thermo Fisher Scientific, Bremen, Germany). 300 ng of peptides were delivered to a trap column (ReproSil-pur C18-AQ, 5 µm, Dr. Maisch, 20 mm × 75 µm, self-packed) at a flow rate of 5 µL/min in 100% solvent A (0.1% formic acid in HPLC grade water). After 10 minutes of loading, peptides were transferred to an analytical column (ReproSil Gold C18-AQ, 3 µm, Dr. Maisch, 400 mm × 75 µm, self-packed) at 50°C and separated using a 60 min gradient from 4% to 32% of solvent B (0.1% FA, 5% DMSO in ACN) in solvent A (0.1% FA, 5% DMSO in HPLC grade water) at 300 nL/min flow rate. The mass spectrometer was operated in data-dependent acquisition and positive ionization mode. MS1 full scans (*m/z* 360–1,300) were acquired with a resolution of 60,000, a normalized AGC value of 100% and maximum injection time of 50 ms. Peptide precursor selection for fragmentation was carried out using a fixed cycle time of 2 s. Only precursors with charge states ranging from 2 to 6 were selected and dynamic exclusion of 30 s was enabled. Peptide fragmentation was performed using higher energy collision induced dissociation (HCD) and a normalized collision energy of 30%. The precursor isolation window width of the quadrupole was set to 1.3 Th. MS2 spectra were acquired with a

resolution of 15,000, a fixed first mass of *m/z* 100, a normalized automatic gain control (AGC) target value of 200% and maximum injection time of 22 ms.

DIA – Vanquish Neo & Exploris 480:

Mixed proteome samples (2 µg/µL protein, prepared in the Tenzer lab, based on the concept initially described in Navarro et al., 2016[1]) containing tryptic digest of human plasma with variable spike-ins of tryptically digested yeast and *E.coli*, respectively, were transferred undiluted to MS vials. Samples were analysed in five replicate injections according to the following measurement scheme: blank-500 ng in-house Hela QC-2x blank-six samples (A9,B9,A3,B3,A1,B1)-blank.

Peptides were analyzed on a Vanquish Neo (micro-flow configuration) coupled on-line to an Orbitrap Exploris 480 mass spectrometer (Thermo Fisher Scientific, Bremen, Germany). 10 µg of peptides were applied onto a commercially available Acclaim PepMap 100 C18 column (2 µm particle size, 1 mm ID × 150 mm, 100 Å pore size; Thermo Fisher Scientific), and separated using a two-stepped gradient: In the first step, a 50 minutes linear gradient ranging from 3% to 24% solvent B (0.1% FA, 3% DMSO in ACN) in solvent A (0.1% FA, 3% DMSO in HPLC grade water) at a flow rate of 50 µL/min was applied. In the second step, solvent B was further increased from 24% to 31% over a 10 minutes linear gradient. The mass spectrometer was operated in data-independent acquisition (DIA) mode. MS1 full scans (*m/z* 360–1,300) were acquired with a resolution of 120,000, a normalized AGC target value of 100% and a maximum injection time of 50 ms. MS2 scans (*m/z* 200–1,800) were acquired from *m/z* 360 to *m/z* 1,260 over a total 40 DIA segments with isolation window widths of 22.5 Th using 1 Th overlap. The scan resolution in the Orbitrap was set to 15,000 with the AGC target set to "standard" and a maximum injection time of 22 ms. The HCD collision energy was set to 28%.

DIA – UltiMate 3000 RSLC & Orbitrap Eclipse:

Mixed proteome samples (2 µg/µL protein, prepared in the Tenzer lab, based on the concept initially described in Navarro et al., 2016[1]) containing tryptic digest of human plasma with variable spike-ins of tryptically digested yeast and *E.coli*, respectively, were diluted 33.3-fold with 0.1% formic acid and transferred to MS vials. Samples were analysed in six replicate injections according to the following measurement scheme: blank-100 ng in-house Hela QC-2x blank-six samples (A9,B9,A3,B3,A1,B1)-blank.

LC-MS/MS measurements were performed on a Dionex Ultimate 3000 RSLC nano system coupled to an Orbitrap Eclipse mass spectrometer (Thermo Fisher Scientific, Bremen, Germany). 300 ng of peptides were delivered to a trap column (ReproSil-pur C18-AQ, 5 µm, Dr. Maisch, 20 mm × 75 µm, self-packed) at a flow rate of 5 µL/min in 100% solvent A (0.1% formic acid in HPLC grade water). After 10 minutes of loading, peptides were transferred to an analytical column (ReproSil Gold C18-AQ, 3 µm, Dr. Maisch, 400 mm × 75 µm, self-packed) at 50°C and separated using a 60 min gradient from 4% to 32% of solvent B (0.1% FA, 5% DMSO in ACN) in solvent A (0.1% FA, 5% DMSO in HPLC grade water) at 300 nL/min flow rate. The mass spectrometer was operated in data-independent acquisition (DIA) mode. MS1 full scans (*m/z* 360–1,300) were acquired with a resolution of 120,000, a normalized AGC target value of 100% and a maximum injection time of 50 ms. MS2 scans (*m/z* 200–1,800) were acquired over a total of 40 DIA segments with variable isolation window widths using 1 Th overlap as listed below:

m/z	z	isolation window (m/z)	m/z	z	isolation window (m/z)
368	2	16	669	2	18
383	2	16	686	2	18
398	2	16	703	2	18
413	2	16	720	2	18
428	2	16	737	2	18
443	2	16	754	2	18
458	2	16	771	2	18
473	2	16	788	2	18
488	2	16	805	2	18
503	2	16	823	2	20
518	2	16	842	2	20
533	2	16	861	2	20
548	2	16	880	2	20
563	2	16	899	2	20
578	2	16	918	2	20
593	2	16	939,5	2	20
608	2	16	963,5	2	25
623	2	16	992,5	2	35
638	2	16	1034	2	55
653	2	16	1179	2	242

The scan resolution in the Orbitrap was set to 30,000 with the AGC target value of 1000% and a maximum injection time of 54 ms. The HCD collision energy was set to 30%.

2.5 Laboratory E

DDA – Ultimate 3000 RSLC & Orbitrap Fusion Lumos:

Mixed proteome samples (2 µg/µL protein, prepared in the Tenzer lab, based on the concept initially described in Navarro et al., 2016[1]) containing tryptic digest of human plasma with variable spike-ins of tryptically digested yeast and *E.coli*, respectively, were diluted 33.3-fold (60 ng/µL protein) with 2% ACN, 0.1% FA in H_2O and transferred to MS vials. Samples for nanoflow measurements were analysed in six replicate injections according to the following measurement scheme: 1. blank, 2. 50 ng Pierce Hela QC, 3. 2x blank, 4. six samples (A9,B9,A3,B3,A1,B1), 5. blank.

LC-MS/MS measurements were performed on a Dionex Ultimate 3000 RSLCnano system coupled to an Orbitrap Fusion Lumos Tribrid mass spectrometer (Thermo Fisher Scientific, Bremen, Germany). 300 ng of peptides were delivered to a trap column (ReproSil-pur C18-AQ, 5 µm, Dr. Maisch, 20 mm × 75 µm, self-packed) at a flow rate of 5 µL/min in 100% solvent A (0.1% formic acid in HPLC grade water). After 10 minutes of loading, peptides were transferred to an analytical column (ReproSil Gold C18-AQ, 3 µm, Dr. Maisch, 400 mm × 75 µm, self-packed) at 50°C and separated using a 60 min gradient from 4% to 32% of solvent B (0.1% FA, 5% DMSO in ACN) in solvent A (0.1% FA, 5% DMSO in HPLC grade water) at 300 nL/min flow rate. The mass spectrometer was operated in data-dependent acquisition and positive ionization mode. MS1 spectra (*m/z* 360–1,300) were recorded at a resolution of 60,000 using an automatic gain control (AGC) value of 4e5 and maximum injection time (maxIT) of 50 ms. MS2 spectra of up to 20 precursor peptides were acquired at a resolution of 15,000 with an automatic gain control (AGC) target value of 5e4 and maximum injection time (maxIT) of 22 ms. The precursor isolation window width was set to 1.3 Th and fragmentation was performed using higher energy collision induced dissociation (HCD) and a normalized collision energy of 30%. Dynamic exclusion was enabled with 20 s exclusion time (mass tolerance +/-10 ppm). Precursors that were singly-charged, unassigned or with charge states >6+ were excluded for fragmentation.

DDA – Vanquish Neo & Exploris 480:

Mixed proteome samples (2 µg/µL protein) were transferred directly into MS vials. Samples were analysed in six replicate injections according to the following

measurement scheme: 1. blank, 2. Pierce Hela QC, 3. 2x blank, 4. six samples (A9,B9,A3,B3,A1,B1), 5. blank.
Peptides were analyzed on a Vanquish Neo (microflow configuration) coupled to an Orbitrap Exploris 480 mass spectrometer (Thermo Fisher Scientific, Bremen, Germany). 10 µg of peptides were applied onto a commercially available Acclaim PepMap 100 C18 column (2 µm particle size, 1 mm ID × 150 mm, 100 Å pore size; Thermo Fisher Scientific), and separated using a two-stepped gradient: In the first step, a 50 minutes linear gradient ranging from 3% to 24% solvent B (0.1% FA, 3% DMSO in ACN) in solvent A (0.1% FA, 3% DMSO in HPLC grade water) at a flow rate of 50 µL/min was applied. In the second step, solvent B was further increased from 24% to 31% over a 10 minutes linear gradient. The mass spectrometer was operated in data-dependent acquisition (DDA) and positive ionization mode. MS1 full scans (*m/z* 360 – 1,300) were acquired with a resolution of 60,000, a normalized AGC target value of 100% and a maximum injection time of 50 ms. Peptide precursor selection for fragmentation was carried out using a fixed cycle time of 1.2 s. Only precursors with charge states ranging from 2 to 6 were selected and dynamic exclusion of 30 s was enabled. Peptide fragmentation was performed using higher energy collision induced dissociation (HCD) and a normalized collision energy of 28%. The precursor isolation window width of the quadrupole was set to 1.1 Th. MS2 spectra were acquired with a resolution of 15,000, a fixed first mass of *m/z* 100, a normalized automatic gain control (AGC) value of 100% and maximum injection time of 40 ms.

<u>DIA – Ultimate 3000 RSLC & Orbitrap Fusion Lumos:</u>
Mixed proteome samples (2 µg/µL protein, prepared in the Tenzer lab, based on the concept initially described in Navarro et al., 2016[1]) containing tryptic digest of human plasma with variable spike-ins of tryptically digested yeast and *E.coli*, respectively, were diluted 33.3-fold (60 ng/µL protein) with 2% ACN, 0.1% FA in H_2O and transferred to MS vials. Samples for nanoflow measurements were analysed in six replicate injections according to the following measurement scheme: 1. blank, 2. 50 ng Pierce Hela QC, 3. 2x blank, 4. six samples (A9,B9,A3,B3,A1,B1), 5. blank.

LC-MS/MS data were acquired on a Dionex Ultimate 3000 RSLCnano system coupled to an Orbitrap Fusion Lumos Tribrid mass spectrometer (Thermo Fisher Scientific, Bremen, Germany). 300 ng of peptides were delivered to a trap column (ReproSil-pur

C18-AQ, 5 µm, Dr. Maisch, 20 mm × 75 µm, self-packed) at a flow rate of 5 µL/min in 100% solvent A (0.1% formic acid in HPLC grade water). After 10 minutes of loading, peptides were transferred to an analytical column (ReproSil Gold C18-AQ, 3 µm, Dr. Maisch, 400 mm × 75 µm, self-packed) at 50°C and separated using a 60 min gradient from 4% to 32% of solvent B (0.1% FA, 5% DMSO in ACN) in solvent A (0.1% FA, 5% DMSO in HPLC grade water) at 300 nL/min flow rate. The mass spectrometer was operated in data-independent acquisition (DIA) mode. MS1 full scans (*m/z* 360 – 1,300) were acquired with a resolution of 120,000, a normalized AGC target value of 1E6 and a maximum injection time of 50 ms. The MS1 scan range covered with DIA windows was set from *m/z* 350 to 1,150 (inclusion list with 41 DIA windows starting at *m/z* 358 to *m/z* 1,111 with variable isolation window widths from 16 Th to 78 Th and 1 Th overlap between scans) as follows:

m/z	z	isolation window (m/z)	m/z	z	isolation window (m/z)
358	2	16	659	2	18
373	2	16	676	2	18
388	2	16	693	2	18
403	2	16	710	2	18
418	2	16	727	2	18
433	2	16	744	2	18
448	2	16	761	2	18
463	2	16	778	2	18
478	2	16	795	2	18
493	2	16	813	2	20
508	2	16	832	2	20
523	2	16	851	2	20
538	2	16	870	2	20
553	2	16	889	2	20
568	2	16	908	2	20
583	2	16	929,5	2	20
598	2	16	953,5	2	25
613	2	16	977,5	2	25
628	2	16	1006,5	2	35
643	2	16	1048	2	50
			1111	2	78

The MS2 scan range was set from *m/z* 200 to *m/z* 1800 at 30,000 resolution with an automatic gain control (AGC) value of 5e5 and a maximum injection time of 54 ms. Fragmentation was performed using higher energy collision induced dissociation (HCD) and a normalized collision energy (NCE) of 30%.

<u>DIA – Vanquish Neo & Exploris 480:</u>

Mixed proteome samples (2 µg/µL protein) were transferred directly into MS vials. Samples were analysed in six replicate injections according to the following

measurement scheme: 1. blank, 2. Pierce Hela QC, 3. 2x blank, 4. six samples (A9,B9,A3,B3,A1,B1), 5. blank.

Peptides were analysed on a Vanquish Neo (microflow configuration) coupled to an Orbitrap Exploris 480 mass spectrometer (Thermo Fisher Scientific, Bremen, Germany). 10 µg of peptides were applied directly onto a commercially available Acclaim PepMap 100 C18 column (2 µm particle size, 1 mm ID × 150 mm, 100 Å pore size; Thermo Fisher Scientific), and separated using two-stepped gradient: In the first step, a 50 minutes linear gradient ranging from 3% to 24% solvent B (0.1% FA, 3% DMSO in ACN) in solvent A (0.1% FA, 3% DMSO in HPLC grade water) at a flow rate of 50 µL/min was applied. In the second step, solvent B was further increased from 24% to 31% over a 10 minutes linear gradient. The mass spectrometer was operated in data-independent acquisition (DIA) mode. MS1 full scans (m/z 360 – 1,300) were acquired with a resolution of 120,000, a normalized AGC target value of 100% and a maximum injection time of 50 ms. The MS1 scan range covered with DIA windows was set from *m/z* 360 to 1,260 using an inclusion list of 40 DIA windows (from *m/z* 371.25 to *m/z* 1248.7) with a fixed isolation window width of 22.5 Th and 1 Th overlap between scans. The MS2 scan range was set from *m/z* 200 to *m/z* 1,800 at 15,000 resolution. Normalized automatic gain control (AGC) target value was used at 100% and a maximum injection time of 22 ms was used. Fragmentation was performed using a normalized collision energy (NCE) of 28%.

2.6 Laboratory F

<u>DDA – Vanquish Neo & Orbitrap Eclipse:</u>

From each sample, 9 µg PYE-peptide mix was injected per run (4.5 µL each). Peptides were separated on a Thermo Scientific™ PepMap™ 100 C18 HPLC Column (1 mm ID x 15 cm lengths) using a Thermo Scientific™ Vanquish Neo UHPLC System. PYE samples were separated using a non-linear 60 min acetonitrile gradient at a flow rate of 50 µL/min (Buffer A: 0.1% FA in H_2O with 3% DMSO, Buffer B: 0.1% FA in ACN with 3% DMSO). The column oven temperature was set to 55°C. Samples were exclusively analysed in data-dependent acquisition (DDA) mode on a Thermo Scientific™ Orbitrap Eclipse™ Tribrid™ mass spectrometer. MS1 scans were acquired using a resolution of 120,000, a scan range of 360-1,300 and a maximum injection time of 50 ms. The cycle time was set to 1.5 s. Charges states from 2-6 were included and the dynamic

exclusion was set to 30 s. MS2 spectra were acquired with the ion trap using a HCD collision energy of 28%. The ion trap scan rate was set to rapid, the maximum injection time was 32 ms, normalized AGC target to 100% and the isolation window to 0.4 Th.

2.7 Laboratory G

DDA – EASY-nLC & timsTOF Pro:

Mixed proteome samples were analyzed on an EASY-nLC 1200 system (Thermo Fisher Scientific, Bremen, Germany) coupled to a timsTOF Pro mass spectrometer with a Captive Spray ion source (Bruker, Bremen, Germany). PYE peptide mixtures (corresponding to 200 ng) were separated on an IonOpticks Aurora column (25 cm x 75 µm, C18 1.6 µm) at a flow rate of 300 nL/min. Column was heated to 50°C. Mobile phase A was ddH_2O (Biosolve Chimie), 0.1% (v/v) FA (Biosolve Chimie), and mobile phase B was 80% ACN in dH_2O, 0.1% (v/v) FA. During the elution, the percentage of solvent B was increased in a linear fashion from 4 to 35% in 30 min, then from 35 to 100% for 10 min. The MS was operated in data-dependent acquisition parallel accumulation-serial fragmentation (ddaPASEF) mode. Full scan MS spectra with mass range m/z 100 to 1700 were acquired. Ion accumulation and separation using trapped ion mobility spectrometry (TIMS) was set to a ramp time of 100 ms with an ion mobility range ($1/K_0$) from 0.85 to 1.30 Vs/cm^2. Number of PASEF ramps was set to 10 with total cycle time of 1.16 s.

DDA - UltiMate 3000 RSLC & Exploris 480:

PYE peptide mixtures (corresponding to 1 µg) were injected to an UltiMate 3000 RSLC nano-HPLC (Dionex, Germering, Germany) coupled to an Exploris 480 mass spectrometer (Thermo Fisher Scientific, Bremen, Germany). Mobile phase A was ddH_2O (Biosolve Chimie), 0.1% (v/v) FA (Biosolve Chimie), and mobile phase B was ACN, 0.1% (v/v) FA. Peptides were separated on a Waters BEH column (75 µm × 250 mm, C18, 1.7 µm, 130 Å) at 35°C running a 48 min linear gradient. The percentage of solvent B was increased in a linear fashion from 2 to 30% in 47 min, then from 30 to 80% for 1 min. After washing the column with 80% B, the column was re-equilibrated to initial conditions resulting in an overall analysis time of 60 min. Data were acquired in a data-dependent mode at a fixed cycle time of 1 s. MS1 spectra were acquired with a resolution of 60,000 covering a mass range from *m/z* 380-1,400

with 300% AGC target and a maximum injection time of 45 ms. The MS2 resolution was set to 15,000 with an AGC target of 100%, and a maximum injection time of 22 ms. Fragment ion data were collected in centroid mode. Normalized HCD collision energy was 26%. First mass was defined at *m/z* 110. Isolation width was set to 1.4 Th.

DIA – EASY-nLC & timsTOF Pro:

Mixed proteome samples were analyzed on an EASY-nLC 1200 system (Thermo Fisher Scientific, Bremen, Germany) coupled to a timsTOF Pro mass spectrometer with a CaptiveSpray ion source (Bruker, Bremen, Germany). PYE peptide mixtures (corresponding to 200 ng) were separated on an IonOpticks Aurora column (25 cm x 75 µm, C18 1.6 µm) at a flow rate of 300 nL/min. Column was heated to 50°C. Mobile phase A was ddH_2O (Biosolve Chimie), 0.1% (v/v) FA (Biosolve Chimie), and mobile phase B was 80% ACN in dH_2O, 0.1% (v/v) FA. During the elution, the percentage of solvent B was increased in a linear fashion from 4 to 35% in 30 min, then from 35 to 100% for 10 min. The MS was operated in data-independent acquisition parallel accumulation-serial fragmentation (diaPASEF) mode. Full scan MS spectra with mass range m/z 100 to 1700 were acquired. Ion accumulation and separation using trapped ion mobility spectrometry (TIMS) was set to a ramp time of 100 ms with an ion mobility range ($1/K_0$) from 0.7 to 1.4 Vs/cm^2. One MS cycle included one TIMS full MS scan followed by multiple MS/MS scans comprising in total 26 windows with a fixed mass width of 30 Da and 0.10 Da overlap covering a *m/z*-range of 374 to 1,152 Da resulting in a cycle time estimate of 1.26 s.

DIA - UltiMate 3000 RSLC & Exploris 480:

PYE peptide mixtures (corresponding to 1 µg) were injected to an UltiMate 3000 RSLC nano-HPLC (Dionex, Germering, Germany) coupled to an Exploris 480 mass spectrometer (Thermo Fisher Scientific, Bremen, Germany). Mobile phase A was ddH_2O (Biosolve Chimie), 0.1% (v/v) FA (Biosolve Chimie), and mobile phase B was ACN, 0.1% (v/v) FA. Peptides were separated on Waters BEH column (75 µm × 250 mm, C18, 1.7 µm, 130 Å) at 35°C running a 48 min linear gradient. The percentage of solvent B was increased in a linear fashion from 2 to 30% in 47 min, then from 30 to 80% for 1 min. After washing the column with 80% B, the column was re-equilibrated to initial conditions resulting in an overall analysis time of 60 min. Data-independent acquisition was performed at a full scan resolution of 120,000. MS1

spectra were recorded over a mass range from *m/z* 350-1,400, maximum injection time was set to 45 ms and the normalized AGC target to 300%. The MS1 scan was followed by 31 DIA scans covering a precursor mass range between *m/z* 410-980 using a variable isolation window widths using the scheme listed below:

m/z	z	isolation window (*m/z*)	m/z	z	isolation window (*m/z*)
410.4365	2	21	663.0513	2	16
429.9453	2	20	678.5584	2	17
447.9535	2	18	694.5656	2	17
464.9612	2	18	711.0731	2	18
481.4688	2	17	729.0813	2	20
496.9758	2	16	747.5897	2	19
512.4828	2	17	766.5984	2	21
527.9899	2	16	786.6074	2	21
542.9967	2	16	807.617	2	23
558.0035	2	16	830.1272	2	24
572.5101	2	15	854.1381	2	26
587.0167	2	16	880.6502	2	29
602.0236	2	16	910.1636	2	32
617.0303	2	16	942.6783	2	35
632.0372	2	16	980.1954	2	42
647.5442	2	17			

The MS2 resolution was set to 30,000 and the AGC target to 1,000%. Normalized HCD collision energy was 28% and fragment ion data were recorded in profile mode.

2.8 Laboratory H

<u>DDA – Ultimate 3000 RSLCnano & Exploris 480:</u>

PYE peptide mixtures (corresponding to 660 ng) were separated on an Ultimate 3000 RSLCnano LC system (Thermo Fisher Scientific, Bremen, Germany) using a Waters BEH reversed-phase column (75 µm × 250 mm, C18, 1.7 µm, 130 Å). Mobile phase A was water containing 0.1% (v/v) formic acid. Peptides were separated running a gradient of 2 to 30% mobile phase B (0.1% (v/v) formic acid in ACN) in 103 min at a flow rate of 300 nL/min. Total analysis time was 120 min including wash and column re-equilibration steps. Column temperature was set to 35°C. Mass spectrometric analysis of eluting peptides was conducted on an Orbitrap Exploris 480 instrument platform (Thermo Fisher Scientific). The funnel RF level was set to 40, and heated capillary temperature was at 275°C.

Data were acquired in data-dependent mode at a fixed cycle time of 2 s. Full MS (MS1) resolution was set to 60,000 at *m/z* 200, full MS automated gain control (AGC) target to 300%, and maximum injection time to 45 ms. Mass range was set to *m/z* 380 –

1,400. The MS2 resolution was set to 15,000 with an AGC target of 100%, and a maximum injection time of 22 ms. Fragment ion data were collected in centroid mode. Normalized HCD collision energy was 26%. Isolation width was set to 1.4 Th. Data were acquired in positive ion mode.

DDA – Ultimate 3000 RSLCnano & Eclipse:

PYE peptide mixtures (corresponding 660 ng) were separated on an Ultimate 3000 RSLCnano LC system (Thermo Fisher Scientific, Bremen, Germany) using a self-packed 30 cm x 100 µm, 1.9 µm reversed-phase column (Reprosil-Pur 120 C18-AQ resin, Dr Maisch). Mobile phase A was water containing 0.1% (v/v) formic acid. Peptides were separated running a gradient of 2 to 38% mobile phase B (0.1% (v/v) formic acid in ACN) in 121 min at a flow rate of 300 nL/min. Total analysis time was 141 min including wash and column re-equilibration steps. Mass spectrometric analysis of eluting peptides was conducted on an Orbitrap Eclipse (Thermo Fisher Scientific).

Data were acquired in data-dependent mode at a fixed cycle time of 3 s. Full MS (MS1) resolution was set to 120,000 at *m/z* 200. Ion transfer time was determined automatically ("auto mode"). Mass range was set to *m/z* 380 – 1,400. The MS2 resolution was set to 15,000 with a maximum injection time of 22 ms. Fragment ion data were collected in centroid mode. Normalized HCD collision energy was 30%. Isolation width was set to 1.2 Th. Data were acquired in positive ion mode.

DIA – Ultimate 3000 RSLCnano & Exploris 480:

PYE peptide mixtures (corresponding to 660 ng) were separated on an Ultimate 3000 RSLCnano LC system (Thermo Fisher Scientific, Bremen, Germany) using a Waters BEH reversed-phase column (75 µm × 250 mm, C18, 1.7 µm, 130 Å). Mobile phase A was water containing 0.1% (v/v) formic acid. Peptides were separated running a gradient of 2 to 30% mobile phase B (0.1% (v/v) formic acid in ACN) in 103 min at a flow rate of 300 nL/min. Total analysis time was 120 min including wash and column re-equilibration steps. Column temperature was set to 35°C. Mass spectrometric analysis of eluting peptides was conducted on an Orbitrap Exploris 480 instrument platform (Thermo Fisher Scientific). The funnel RF level was set to 40, and heated capillary temperature was at 275°C.

Data were acquired in DIA mode, full MS (MS1) resolution was set to 120,000 at *m/z* 200 and full MS automated gain control (AGC) target to 300% with a maximum injection time of 45 ms. Mass range was set to *m/z* 350–1,400. Fragment ion spectra were acquired with an AGC target value of 1000%, applying a DIA scheme consisting of 47 windows with variable window widths and 1 Th overlap. MS2 resolution was set to 30,000 and maximum injection time was set to 54 ms. Normalized collision energy was fixed at 28% and fragment ion data collected in centroid mode. Data were acquired in positive ion mode.

<u>DIA – Ultimate 3000 RSLCnano & Eclipse:</u>

PYE peptide mixtures (corresponding 660 ng) were separated on an Ultimate 3000 RSLCnano LC system (Thermo Fisher Scientific, Bremen, Germany) using a self-packed 30 cm x 100 µm, 1.9 µm reversed-phase column (Reprosil-Pur 120 C18-AQ resin, Dr Maisch). Mobile phase A was water containing 0.1% (v/v) formic acid. Peptides were separated running a gradient of 2 to 38% mobile phase B (0.1% (v/v) formic acid in ACN) in 121 min at a flow rate of 300 nL/min. Total analysis time was 141 min including wash and column re-equilibration steps. Column temperature was set to 35°C. Mass spectrometric analysis of eluting peptides was conducted on an Orbitrap Eclipse (Thermo Fisher Scientific).

Data were acquired in DIA mode, full MS (MS1) resolution was set to 120,000 at *m/z* 200 and full MS automated gain control (AGC) target to 300% with a maximum injection time of 45 ms. Mass range was set to *m/z* 350–1,400. Fragment ion spectra were acquired applying a DIA scheme consisting of 55 windows with variable window widths and 1 Th overlap. MS2 resolution was set to 30,000 and maximum injection time was set to 54 ms. Normalized collision energy was fixed at 28% and fragment ion data collected in profile mode. Data were acquired in positive ion mode.

2.9 Laboratory I

<u>DIA – EASY-nLC & Exploris 480:</u>

HeLa QC standard samples as well as blanks were acquired in data-dependent mode. An EASY-nLC 1200 (Thermo Fisher Scientific, Bremen, Germany) was coupled to an Orbitrap Exploris 480 mass spectrometer (Thermo Fisher Scientific). Samples were separated using a 44 min gradient and loaded directly onto the analytical column

(75 µm x 20 cm column packed in-house with C18-AQ 1.9 µm beads, Dr. Maisch Reprosil-Pur 120) at a flow rate of 250 nL/min. A total amount of 100 ng HeLa Protein Digest Standard (Pierce) was injected on column to perform a system QC. MS1 was recorded at a resolution of 60,000 and MS2 at 15,000. The top20 abundant peaks were selected for fragmentation using a 1.3 Th isolation window and 22 ms maximum injection time with an AGC target set to 100% corresponding to 100,000 ions.

DIA acquisition of the main sample cohort (PYE peptide mixtures) was done using the same LC-MS setup as described above. MS1 spectra were acquired at a resolution of 120,000 and MS2 at a resolution of 30,000. A number of 40 isolation windows with variable isolation widths were used for data-independent acquisition mode. The AGC target was set to 3,000% corresponding to a number of 3e6 ions and the maximum injection time was specified to 54 ms. For fragmentation, a stepped collision energy approach was used.

2.10 Laboratory J

DIA – EASY-nLC & timsTOF Pro2:

The samples were measured on an EASY-nLC 1200 system (Thermo Fisher Scientific, Bremen, Germany) coupled to a TIMS quadrupole TOF mass spectrometer (Bruker timsTOF Pro2). Peptides were separated using a 44 min gradient at a flow rate of 250 nL/min on a home-packed column (20 cm × 75 µm i.d., 1.9 µm ReproSil-Pur C18-AQ particles from Dr. Maisch) at 50℃.

To perform analyses in diaPASEF mode, the default method from Bruker was used (‘diaPASEF – long gradient’) with a scan range of *m/z* 100 to 1,700, ion mobility range of 0.6-1.6 $1/K_0$ and a dia window scheme comprising 32 windows with a fixed window width of 26 Th and 1 Th overlap covering a precursor *m/z*-range from 400-1,201. The tims ramp time was set to 100 ms resulting in an overall cycle time of approx. 1.7 s.

2.11 Laboratory K

DIA – Acquity M-Class & ZenoTOF 7600:

Samples were acquired in data-independent acquisition (DIA)/Zeno-SWATH mode, on a ZenoTOF 7600 mass spectrometer (SCIEX, Toronto, Canada) coupled online to an

Acquity M-Class UPLC system (Waters, Milford, MA, USA). Each measurement used 0.5 µg sample which was separated by reversed-phase chromatography (on a nanoEase M/Z HSS T3 column; 100 Å, 1.8 µm, 0.3 x 150 mm) with buffers A (0.1% formic acid) and B (acetonitrile with 0.1% formic acid) ramping from 3 to 40% buffer B at a flow rate of 5 µL/min and at 35°C using a 30 min gradient as described by Wang *et al.*[2].
The mass spectrometric method was identical to the one employed by Wang *et al.*[2] with the following exceptions: The spray voltage was set to 5,000 V and a scheduled ionization between minutes 2.5 and 22 was used.

2.12 Laboratory L

DIA - nanoAcquity & timsTOF Pro:

Samples (corresponding to 250 ng) were analyzed on a timsTOF-Pro (Bruker Corporation, Billerica, MA, USA) coupled online to a nanoAcquity LC system (Waters). Peptides were directly injected onto an Aurora 5 cm x 150 µm analytical emitter column (1.6 µm C18, IonOpticks, Australia) at a flow rate of 2 µL/min for loading, washing and equilibration, and 1 µL/min for the separation of peptides running a linear gradient from 0 to 34% B (mobile phase A = 0.1% FA in water, mobile phase B = 0.1% FA in ACN). Eluting peptides were analyzed in positive ion mode using parallel accumulation serial fragmentation (PASEF) enhanced DIA. The dual TIMS (trapped ion mobility spectrometer) was operated at a fixed duty cycle close to 100% using equal accumulation and ramp times of 50 ms each spanning a mobility range from $1/K_0$ = 0.6 Vs cm^{-2} to 1.4 Vs cm^{-2}. We defined 36 × 25 Th isolation windows with 1 Th overlap covering a precursor mass range from *m/z* 300 to 1,165 resulting in 2-3 diaPASEF scans per TIMS cycle and an overall cycle time of 0.84 s. The collision energy was ramped linearly as a function of the mobility from 59 eV at $1/K_0$ = 1.3 Vs cm^{-2} to 20 eV at $1/K_0$ = 0.85 Vs cm^{-2}. MS1 and fragment ion spectra were recorded with a mass range spanning from *m/z* 100-1,700.

DIA - nanoElute & timsTOF Pro2:

PYE peptide mixtures (corresponding to 250 ng) were injected and separated on a nanoElute LC system (Bruker Corporation, Billerica, MA, USA) at a flow rate of 850 nL/min using a reversed phase C18 column (PepSep™, 25 cm x 150 µm 1.5 µm)

which was heated to 40°C. Peptides were loaded onto the column in direct injection mode at 800 bar. Mobile phase A was 0.1% FA (v/v) in water and mobile phase B 0.1% FA (v/v) in ACN. Peptides were separated running a non-linear gradient from 2% to 38% mobile phase B over 35.5 min. Afterwards, column was rinsed for 5.5 min at 95% B. Eluting peptides were analyzed in positive mode ESI-MS using parallel accumulation serial fragmentation (PASEF) enhanced DIA mode on a timsTOF Pro2 mass spectrometer (Bruker Corporation). The dual TIMS was operated at a fixed duty cycle close to 100% using equal accumulation and ramp times of 100 ms each spanning a mobility range from $1/K_0$ = 0.6 Vs cm^{-2} to 1.6 Vs cm^{-2}. We defined 36 × 25 Th isolation windows from *m/z* 300 to 1,165 resulting in 2-3 diaPASEF scans per TIMS cycle and an overall cycle time of 1.7 s. The collision energy was ramped linearly as a function of the mobility from 59 eV at $1/K_0$ = 1.3 Vs cm^{-2} to 20 eV at $1/K_0$ = 0.85 Vs cm^{-2}. MS1 and fragment ion spectra were recorded with a mass range spanning from *m/z* 100-1,700.

DIA – Ultimate 3000 RSLCnano & Exploris 480:

PYE peptide mixtures (corresponding to 150 ng) were separated on an Ultimate 3000 RSLCnano LC system (Thermo Fisher Scientific, Bremen, Germany) using a PEPMAP100 C18 5 µm 0.3 x 5 mm trap (Thermo Fisher Scientific) and an HSS-T3 C18 1.8 µm, 100 µm x 100 mm analytical reversed-phase column (Waters Corporation). Mobile phase A was water containing 0.1% (v/v) formic acid and 3% (v/v) DMSO. Peptides were separated running a gradient of 2 to 35% mobile phase B (0.1% (v/v) formic acid, 3% (v/v) DMSO in ACN) in 29 min at a flow rate of 800 nL/min. Total analysis time was 40 min including wash and column re-equilibration steps. Column temperature was set to 55°C. Mass spectrometric analysis of eluting peptides was conducted on an Orbitrap Exploris 480 instrument platform (Thermo Fisher Scientific). The funnel RF level was set to 40, and heated capillary temperature was at 250°C. Data were acquired in DIA mode, full MS (MS1) resolution was set to 120,000 at *m/z* 200 and full MS automated gain control (AGC) target to 300%. Mass range was set to *m/z* 345–1,250. Fragment ion spectra were acquired with an AGC target value of 1000%, applying a DIA scheme consisting of 21 windows (with a variable window width and a 0.5 Th window overlap). Resolution was set to 30,000 and ion transfer time was determined automatically ("auto mode"). Normalized collision energy was fixed at 27%. Data were acquired in positive ion mode.

DIA – Ultimate 3000 RSLCnano & Exploris 480 with FAIMS:

PYE peptide mixtures (corresponding to 150 ng) were separated on an Ultimate 3000 RSLCnano LC system (Thermo Fisher Scientific, Bremen, Germany) using a PEPMAP100 C18 5 µm 0.3 x 5 mm trap (Thermo Fisher Scientific) and an HSS-T3 C18 1.8 µm, 100 µm x 100 mm analytical reversed-phase column (Waters Corporation). Mobile phase A was water containing 0.1% (v/v) formic acid and 3% (v/v) DMSO. Peptides were separated running a gradient of 2 to 35% mobile phase B (0.1% (v/v) formic acid, 3% (v/v) DMSO in ACN) in 29 min at a flow rate of 800 nL/min. Total analysis time was 40 min including wash and column re-equilibration steps. Column temperature was set to 55°C. Mass spectrometric analysis of eluting peptides was conducted on an Orbitrap Exploris 480 instrument platform coupled to a FAIMS Pro interface (Thermo Fisher Scientific). The funnel RF level was set to 40, and heated capillary temperature was at 275 °C.

Data were acquired in DIA mode, full MS (MS1) resolution was set to 120,000 at *m/z* 200 and full MS automated gain control (AGC) target to 300%. Mass range was set to *m/z* 345–1,250. Fragment ion spectra were acquired with an AGC target value of 1000%, applying a DIA scheme consisting of 20 windows (window width: 45 Th with a 0.5 Th overlap). Resolution was set to 30,000 and ion transfer time was determined automatically (“auto mode”). Normalized collision energy was fixed at 27%. Data were acquired in positive ion mode. FAIMS voltage was set to -36 V.

REFERENCES

1. Navarro, P., Kuharev, J., Gillet, L.C., Bernhardt, O.M., MacLean, B., Röst, H.L., Tate, S.A., Tsou, C.-C., Reiter, L., Distler, U., et al. (2016). A multicenter study benchmarks software tools for label-free proteome quantification. Nat Biotechnol *34*, 1130–1136. https://doi.org/10.1038/nbt.3685.
2. Wang, Z., Mülleder, M., Batruch, I., Chelur, A., Textoris-Taube, K., Schwecke, T., Hartl, J., Causon, J., Castro-Perez, J., Demichev, V., et al. (2022). High-throughput proteomics of nanogram-scale samples with Zeno SWATH MS. Elife *11*. https://doi.org/10.7554/ELIFE.83947.
