# Supplementary material for: Multicenter evaluation of label-free quantification in human plasma on a high dynamic range benchmark set
Source: Nat Commun. 2025 Oct 2;16:8774. doi: 10.1038/s41467-025-64501-z (PMC12491457; doi:10.1038/s41467-025-64501-z)
Supplement: Supplementary file 2 — Description of Additional Supplementary Files [file 41467_2025_64501_MOESM2_ESM.pdf]

Description of Additional Supplementary Files

File Name: Supplementary Data 1

Description: Overview of LC-MS setups used in the PYE ring trial.

File Name: Supplementary Data 2

Description: Numbers of identified peptides and proteins for each experimental LC-MS setup in PYE1.

File Name: Supplementary Data 3

Description: Numbers of identified proteins for each experimental LC-MS setup in PYE1, PYE3 and PYE9 split by species, i.e., human, yeast and *E. coli*.

File Name: Supplementary Data 4

Description: Summary of coefficients of variation (CVs) of protein abundances in samples PYE1 A and PYE1 B for the 14 DDA and 20 DIA datasets analysed by MaxQuant and DIA-NN, respectively. Additionally, data displayed in figure panels Fig. 4 e,f are summarized including retention times (RT) CVs and peak capacities for the DDA and DIA data.

File Name: Supplementary Data 5

Description: Summary of precision and accuracy values for the 13 mapping DDA and DIA datasets. Calculated log_2(FC) values (median value of the distribution) between samples PYE1 A and B are listed in the present table along with the global precision of quantification defined by the interquartile range and the standard deviation of the log_2(FC) distribution of human, yeast and *E. coli* proteins.

File Name: Supplementary Data 6

Description: Summary of precision and accuracy values for the PYE1, PYE3 and PYE9 samples analysed with 20 different DIA LC-MS setups. Calculated log_2(FC) values (median value of the distribution) between samples A and B are listed in the present table along with the global precision of quantification defined by the interquartile range and the standard deviation of the log_2(FC) distribution of human, yeast and *E. coli* proteins.

File Name: Supplementary Data 7

Description: Summary of different metrics for yeast proteins identified in the DIA dataset in sample sets PYE1, PYE3 and PYE9, such as protein ID number, precision and accuracy values, number of datapoints at FWHM as reported in DIA-NN (DP_FWHM).

File Name: Supplementary Data 8

Description: MaxQuant and DIA-NN processing parameters.

File Name: Supplementary Data 9

Description: Overview and index of all files (raw data and search results) uploaded to jPOST/Proteomeexchange with the following identifiers: PXD056598 for ProteomeXchange and JPST003358 for jPOST.
