# Supplementary material for: Multicenter evaluation of label-free quantification in human plasma on a high dynamic range benchmark set
Source: Nat Commun. 2025 Oct 2;16:8774. doi: 10.1038/s41467-025-64501-z (PMC12491457; doi:10.1038/s41467-025-64501-z)
Supplement: Supplementary file 12 — Reporting Summary [file 41467_2025_64501_MOESM12_ESM.pdf]

nature portfolio

Corresponding author(s): Ute Distler
Stefan Tenzer

Last updated by author(s): Sep 1, 2025

Reporting Summary

Nature Portfolio wishes to improve the reproducibility of the work that we publish. This form provides structure for consistency and transparency in reporting. For further information on Nature Portfolio policies, see our Editorial Policies and the Editorial Policy Checklist.

Statistics

For all statistical analyses, confirm that the following items are present in the figure legend, table legend, main text, or Methods section.

n/a	Confirmed	
☐	☒	The exact sample size (*n*) for each experimental group/condition, given as a discrete number and unit of measurement
☐	☒	A statement on whether measurements were taken from distinct samples or whether the same sample was measured repeatedly
☒	☐	The statistical test(s) used AND whether they are one- or two-sided<br>*Only common tests should be described solely by name; describe more complex techniques in the Methods section.*
☐	☒	A description of all covariates tested
☐	☒	A description of any assumptions or corrections, such as tests of normality and adjustment for multiple comparisons
☐	☒	A full description of the statistical parameters including central tendency (e.g. means) or other basic estimates (e.g. regression coefficient) AND variation (e.g. standard deviation) or associated estimates of uncertainty (e.g. confidence intervals)
☒	☐	For null hypothesis testing, the test statistic (e.g. *F*, *t*, *r*) with confidence intervals, effect sizes, degrees of freedom and *P* value noted<br>*Give P values as exact values whenever suitable.*
☒	☐	For Bayesian analysis, information on the choice of priors and Markov chain Monte Carlo settings
☒	☐	For hierarchical and complex designs, identification of the appropriate level for tests and full reporting of outcomes
☒	☐	Estimates of effect sizes (e.g. Cohen's *d*, Pearson's *r*), indicating how they were calculated

Our web collection on statistics for biologists contains articles on many of the points above.

Software and code

Policy information about availability of computer code

Data collection	na
Data analysis	Raw data processing and label-free quantification of mass spectrometric datasets was performed in MaxQuant (version 2.3.1.0; DDA data) and in DIA-NN (version 1.8.1; DIA data). PYE raw data were additionally processed using FragPipe (version 23.0, DDA and DIA data). For FragPipe processing, ZenoTOF raw files were converted to mzML using MSConvert (version 3.0.20280).<br>R (version 4.3.2) was used to integrate datasets and plot the data. In-house R scripts used and were based on published packages including the mpwR package (https://CRAN.R-project.org/package=mpwR), the LFQBench package (https://github.com/IFIproteomics/LFQbench), ggplot2 (https://ggplot2.tidyverse.org/) and 'ComplexUpset' (https://krassowski.github.io/complex-upset/). No novel algorithms were developed for data analysis.<br>The R scripts for reproducing the figures are available via GitHub at [https://github.com/HanYoo1402/LFQ-Bench-Scripts-for-PYE-Multicenter-Study] and Zenodo at [https://doi.org/zenodo.org/records/17018339].<br>Figure subpanels have been integrated using Adobe Illustrator (version 29.7.1). Bar plots in Figs. 1 and 7 have been generated using GraphPad Prism (version 10.5.0).

For manuscripts utilizing custom algorithms or software that are central to the research but not yet described in published literature, software must be made available to editors and reviewers. We strongly encourage code deposition in a community repository (e.g. GitHub). See the Nature Portfolio guidelines for submitting code & software for further information.

Data

Policy information about availability of data

All manuscripts must include a data availability statement. This statement should provide the following information, where applicable:

- Accession codes, unique identifiers, or web links for publicly available datasets
- A description of any restrictions on data availability
- For clinical datasets or third party data, please ensure that the statement adheres to our policy

The raw mass spectrometry data generated in this study along with the database search results have been deposited to the ProteomeXchange Consortium (http://proteomecentral.proteomexchange.org) via the jPOST partner repository with the dataset identifiers PXD056598 (ProteomeXchange) [https://proteomecentral.proteomexchange.org/cgi/GetDataset?ID=PXD056598] and JPST003358 (jPOST, https://repository.jpostdb.org/entry/JPST003358) (PYE analyses from all partner sites as well as plasma proteome experiments).
Source data are provided with this paper via Zenodo at [https://doi.org/10.5281/zenodo.16994622]. Additional data files providing a full summary of identified proteins and peptides across all sites for the DDA and DIA analyses can be also assessed via Zenodo at [https://doi.org/10.5281/zenodo.16994622].

Research involving human participants, their data, or biological material

Policy information about studies with human participants or human data. See also policy information about sex, gender (identity/presentation), and sexual orientation and race, ethnicity and racism.

Reporting on sex and gender	na
Reporting on race, ethnicity, or other socially relevant groupings	na
Population characteristics	na
Recruitment	Human plasma pool was commercially obtained from BioCat GmbH (Heidelberg, Germany).
Ethics oversight	Blood samples for revision measurements (Fig. 7 f,g) were taken at the University Medical Centre of the Johannes Gutenberg University Mainz from five healthy donors after obtaining informed consent. All experiments containing human blood plasma from these donors were approved by the ethics committee of the Landesärztekammer Rheinland-Pfalz, Mainz No. 837.439.12 (8540-F) and thus performed in compliance with all relevant laws and guidelines.

Note that full information on the approval of the study protocol must also be provided in the manuscript.

Field-specific reporting

Please select the one below that is the best fit for your research. If you are not sure, read the appropriate sections before making your selection.

[x] Life sciences [ ] Behavioural & social sciences [ ] Ecological, evolutionary & environmental sciences

For a reference copy of the document with all sections, see nature.com/documents/nr-reporting-summary-flat.pdf

Life sciences study design

All studies must disclose on these points even when the disclosure is negative.

Sample size	Synthetic Benchmark Samples
Data exclusions	One data file had to be excluded: C_nE_tTOF PYE3 B replicate 1 DIA. Here, the number of identifications in DIA-NN were below 60% of average as compared to the remaining replicates of this sample and setup.<br>Beside this file, we did not exclude any data from the analysis. Table 1 and Supplementary Data 1 summarize the number of replicate injections acquired for each LC_MS setup<br>In the footnote of Table 1 we indicate that one raw file had to be removed.
Replication	Mulit-center, multi-instrument benchmark
Randomization	na
Blinding	na

Reporting for specific materials, systems and methods

We require information from authors about some types of materials, experimental systems and methods used in many studies. Here, indicate whether each material, system or method listed is relevant to your study. If you are not sure if a list item applies to your research, read the appropriate section before selecting a response.

Materials & experimental systems

n/a	Involved in the study
[x]	[ ] Antibodies
[x]	[ ] Eukaryotic cell lines
[x]	[ ] Palaeontology and archaeology
[x]	[ ] Animals and other organisms
[x]	[ ] Clinical data
[x]	[ ] Dual use research of concern
[x]	[ ] Plants

Methods

n/a	Involved in the study
[x]	[ ] ChIP-seq
[x]	[ ] Flow cytometry
[x]	[ ] MRI-based neuroimaging

Plants

Seed stocks	na
Novel plant genotypes	na
Authentication	na
